# Genomic and evolutionary characteristics of metastatic gastric cancer by routes

**DOI:** 10.1038/s41416-023-02338-3

**Published:** 2023-07-08

**Authors:** Jae Eun Lee, Ki Tae Kim, Su-Jin Shin, Jae-Ho Cheong, Yoon Young Choi

**Affiliations:** 1 Portrai Inc., Seoul, Korea; 2 Department of Surgery, Yonsei University Health System, Yonsei University College of Medicine, Seoul, South Korea; 3 Department of Molecular Genetics & Dental Pharmacology, School of Dentistry, Seoul National University, Seoul, South Korea; 4 Dental Research Institute and Dental Multi-omics Center, Seoul National University, Seoul, South Korea; 5 Department of Pathology, Yonsei University Health System, Yonsei University College of Medicine, Seoul, South Korea; 6 Department of Surgery, Soonchunhyang Bucheon Hospital, Soonchunhyang University College of Medicine, Bucheon, South Korea

**Keywords:** Gastric cancer, Cancer genomics

## Abstract

**Background:**

In gastric cancer (GC) patients, metastatic progression through the lymphatic, hematogenous, peritoneal, and ovarian routes, is the ultimate cause of death. However, the genomic and evolutionary characteristics of metastatic GC have not been widely evaluated.

**Methods:**

Whole-exome sequencing data were analyzed for 99 primary and paired metastatic gastric cancers from 15 patients who underwent gastrectomy and metastasectomy.

**Results:**

Hematogenous metastatic tumors were associated with increased chromosomal instability and de novo gain/amplification in cancer driver genes, whereas peritoneal/ovarian metastasis was linked to sustained chromosomal stability and de novo somatic mutations in driver genes. The genomic distance of the hematogenous and peritoneal metastatic tumors was found to be closer to the primary tumors than lymph node (LN) metastasis, while ovarian metastasis was closer to LN and peritoneal metastasis than the primary tumor. Two migration patterns for metastatic GCs were identified; branched and diaspora. Both molecular subtypes of the metastatic tumors, rather than the primary tumor, and their migration patterns were related to patient survival.

**Conclusions:**

Genomic characteristics of metastatic gastric cancer is distinctive by routes and associated with patients’ prognosis along with genomic evolution pattenrs, indicating that both primary and metastatic gastric cancers require genomic evaluation.

## Background

Cancer metastasis is the ultimate cause of death in cancer patients; thus, it is crucial that we understand its clinical and biological characteristics. Recent advances in sequencing technologies have improved our understanding of cancer at the genomic level, and the molecular characteristics of primary tumors have been thoroughly evaluated, which has led to the current levels of precision in oncology [1–3]. Further studies on metastatic cancers have identified unique alterations in metastatic tumors that could be possible targets to improve patient survival [4], and genomic alterations driving cancer metastasis were found to be different based on the type of primary tumor [3].

**Fig. 1:**
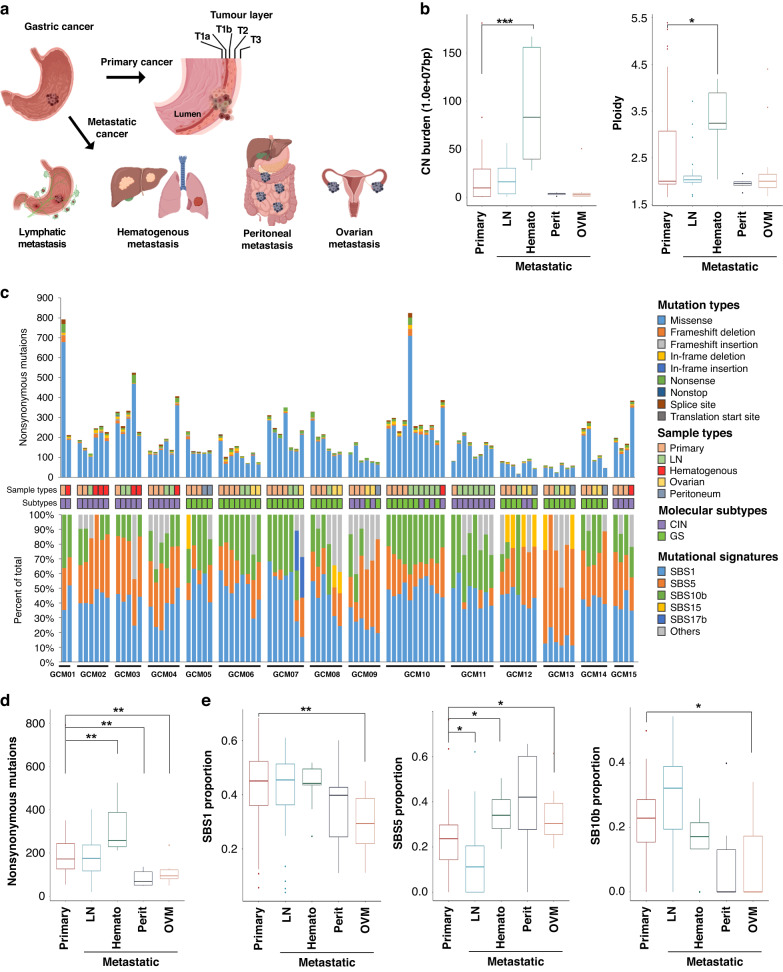
Genomic architectures of the different metastatic gastric cancers and their routes. **a** Schematic illustrating the various metastatic routes of gastric cancer. As the cancer grows, it invades the wall of the stomach and then spreads through the lymphatics, blood, and peritoneal space causing various types of metastases. **b** Comparison of the copy number burden and ploidy (y-axis) between primary and metastatic tumors by route (x-axis). Higher copy number burdens and ploidies were identified in hematogenous metastasis. **c** Summary of the number and types of mutation in each case and the mutational signatures in each tumor and patient. **d** Comparison of the number of nonsynonymous mutations (y-axis) between primary and metastatic tumors by route (x-axis). Compared to the primary tumors, the number was higher in hematogenous, but lower in peritoneal and ovarian metastasis. **e** Comparison of the proportions for each dominant signature (y-axis) between the primary and metastatic tumors by route (x-axis). *, **, and ***, indicate *P* < 0.05, <0.005 and <0.001 using the Mann–Whitney test, respectively.

Gastric cancer (GC) is one of the most common and lethal cancers in the world [5, 6]. Since the stomach is supplied with blood by five main vessels, (the right and left gastric, the right and left gastro-epiploic, and short gastric arteries) the venous and lymphatic drainage of the stomach is complicated. In addition, cancer cells can spread to the peritoneal space through the outer layer of the stomach. These anatomical characteristics of the stomach make the pattern of GC metastasis distinctive from that of other cancer types; peritoneal metastasis is the most common, ovarian metastasis (also called Krukenberg tumor) mainly develops from GC, and the extent and location of lymph node (LN) metastasis is difficult to predict (Fig. 1a) [7–9]. In the clinical practice, LNs around the stomach are classified using a tier system based on their anatomical locations: perigastric (D1), extra-perigastric (D2), and distant (D4 level) [10]. It is assumed that LN metastasis is sequential from proximal (D1) to distal (D2/D4) [11].

Evolutionarily, cancer cells acquire metastatic potential through random mutations, genetic drift, and non-random selection [12]. Consequently, primary and metastatic cancer cells will be genetically heterogeneous, and this is the rational basis for phylogenetic analysis. To date, genomic heterogeneity between primary and metastatic tumors and their phylogenetic evolution in gastric cancer has been reported [13–15], however, comparison by metastatic routes has not been widely evaluated. In addition, considering that the molecular subtypes of GC are associated with distinct histologic and clinical characteristics, including the route of metastasis [2, 16], genomic alterations could be similarly affected, though this has not yet been widely evaluated.

Here, we analyzed whole-exome sequencing data for 99 primary and paired-multiregional metastatic GCs from 15 patients who underwent gastrectomy with metastasectomy. We compared the genomic characteristics of the metastatic GC based on the route. We identified that the genomic alterations driving GC metastasis differed in their routes and that the metastatic GC subtype was more important than the primary tumor. We also reconstructed phylogenetic trees and identified two metastatic migration patterns: branched and diaspora progression; these were associated with patient survival. Moreover, the migration history and genomic distance of the LN metastasis of GC suggested that it is an independent event rather than a sequential event, regardless of the anatomical location.

## Methods

### Tissue samples

#### Patients and tissue samples

We reviewed the data for 7430 patients who underwent gastrectomy with lymphadenectomy for GC in Severance Hospital, Seoul, Korea from January 2006 to December 2012. Patients were selected if they met the following criteria: 1) pathologically confirmed gastric adenocarcinoma; 2) synchronous or metachronous metastatic GC and received metastasectomy; 3) metastatic cancer that included hematogenous (liver or lung), peritoneum, ovary, or distant LNs (para-aortic-#16 or superior mesenteric vein-#14v); and 4) formalin-fixed paraffin-embedded (FFPE) tissues were available. The LNs were presented as a number based on their anatomical location and classified using a tier system as follows: D1 (LNs at perigastric level, #1–7), D2 (LNs at extra-perigastric level, #8–12), and D4 (distant LNs, #14v, 16) [10]. Clinically, D1 and D2 levels are considered regional LNs and D4 level are considered distant metastasis. This study was approved by the Institutional Review Board (IRB) of Severance Hospital (4-2019-0188), and informed consent was received for all patients. The patients had been treated following the gastric cancer treatment guidelines [17, 18]. The clinical demographics of the enrolled patients and samples are described in Supplementary Tables 1 and 2.

#### Whole-exome sequencing (WES)

For tissue sequencing, the FFPE of collected surgical specimens that were histologically confirmed as tumor samples were used, and normal gastric mucosa samples were used as the control. The histology of all samples was reviewed by a GC specialized pathologist (SS), and genomic DNA was obtained by macro-dissection of serial unstained sections from the tumor-enriched area. Multi-regional WES was conducted for the primary tumors. They were separated by layer as T1a/b, T2, and T3 to represent the mucosa/submucosa, proper muscle, and subserosa layer of the tumor according to the pathologic T stage of the GC, respectively [19]. SureSelect sequencing libraries were prepared following the manufacturer’s instructions (Agilent SureSelect All Exon V6 kit, SantaClara, CA, USA), and the NovaSeq 6000 sequencing system (Illumina^TM^, San Diego, CA, USA) was used to conduct sequencing with read lengths of 2 ×100 bp and a mean depth of 142.4× (range from 49× to 203×). The statistics and quality metrics for the normal and tumor samples are described in Supplementary Tables S3 and S4. Paired-end sequencing data were aligned to the human reference genome (hg19) using the Burrows–Wheeler Aligner (BWA 0.7.17) algorithm. The removal of duplicated reads, base quality recalibration, and multiple sequence realignment were all conducted using the Picard and Genome Analysis Toolkit (GATK v3.9) with bcbio-nextgen (v.1.2.3) [20]. For somatic analysis, a paired-normal sequence was used as a reference and point mutation and small insertions and deletions were detected using Mutect2 (v2.2), and variants were annotated using VEP (ENSEMBL’s Variant Effect Predictor v99) [21].

#### Filtering of variants

Somatic variants were filtered following GATK best practice [22], and variants were selected with TLOD ≥ 10 & MMQ ≥ 60 & SEQQ ≥ 20 & STRANDQ ≥ 20. Additional filtering was applied using the following criteria in each patient: variants with patient-matched normal coverage of ≥10 reads and zero variant counts, tumor coverage ≥20 in all tumor samples, and a variant allele frequency ≥0.05 in at least one tumor sample.

#### Copy number and molecular subtype classification of the tumors

The copy number (CN) alterations for the tumor were compared to those of the paired-normal that were estimated using the CNVkit software (v0.9.8) [23]. CN burden was calculated as the sum of the bp from the segments with a |CN| > 0.3 in each sample. Gain and amplification (gain/amp) at the gene level were evaluated for over one and four CN changes. The microsatellite status of each sample was estimated using MSIsensor-pro (v1.2.0) [24], and scores >5 were considered to indicate microsatellite instability (MSI)-high. The Epstein–Barr virus (EBV) reference sequence from the Genomic Data Commons was checked in each tumor sequence to define the EBV type GC.

GC is classified into four-molecular subtypes by The Cancer Genome Atlas (TCGA); [2] microsatellite unstable (MSI-H) and EBV type and chromosomal instability (CIN) and genomic stable (GS), which are divided according to the CN burden. The subtypes of the primary and metastatic tumors in this cohort were classified using the same approach as in TCGA; MSI-H, and EBV related tumors, and for non-MSI/EBV tumors, CIN and GS subtypes were classified using CN based clustering, Euclidean distances using Ward’s method along with the TCGA of the GC (STAD) samples (*n* = 440, level 3 data). The chromosomal arms were considered altered if at least 66% of the arm was lost or gained with a log2 CN greater than 0.1. The genome-wide total and allele-specific CN with sample purity were estimated using the ASCAT algorithm (v2.5.2) [25].

#### Mutational signatures

Mutational signatures were estimated using the method described by Alexandrov et al. [26] with SigProfilerExtractor (v1.1.4) using the default option [27]. Statistical comparisons of the major signatures were conducted by the origin of tumors. The samples with >50% sequencing artifact related signatures, like SBS 49, were excluded from further mutation related analysis including phylogenetic analysis.

#### Phylogenetic analysis

Treeomics v1.9.0 [28] and Minimum Event Distance for Intra-tumor Copy-number Comparisons 2 (MEDICC2 v0.7.0) [29] were used with the default settings to reconstruct the phylogenies of the metastatic tumors using high-quality somatic variants and the CN alterations were identified by WES, respectively. Treeomics uses a Bayesian inference model to account for sequencing errors and low purity and it employs Integer Linear Programming to infer a maximum likelihood tree. The genetic distance and Jaccard similarity coefficient between all pairs of samples in each patient were calculated using Treeomics. MEDICC2 is a phylogeny inference algorithm for allele-specific somatic copy number alteration (SCNA) data that addresses the chromosomal instability and SCNAs including whole genome doubling (WGD). Considering the phylogeny, three researchers (JEL, KTK, YYC) decided on two types of consensus metastatic patterns: branched and diaspora progression. The branched progression has a long trunk, which harbors the initiating cancer driver alterations, indicating that a founder clone could acquire driver alterations and disseminate late from the primary tumor to evolve into a metastatic a tumor. The diaspora pattern has a short trunk, and the phylogenetic tree involved multiple branches form a founder clone, giving each metastatic tumor distinct driver alterations. This indicates that the metastatic subclone has not yet acquired driver gene alterations that were disseminated from the primary tumors early and that they have evolved independently of each other, resulting in substantial genetic divergence between the primary and metastatic tumors [30, 31].

#### Migration pattern inferences

To infer the parsimonious migration history of metastatic tumors, especially for LN metastasis from primary tumors of GC, we used PyClone (0.13.0) [32] and Metastatic And Clonal History INtegrative Analysis (MACHINA v1.2) [33]. The resulting mutations and associated major and minor CN were clustered with PyClone using default settings, and the clusters with >5 variants were selected. The PyClone consensus cluster files were used to enumerate evolutionary relationships, and the combination of PyClone cluster frequency estimates and enumerated trees were used to search for the most parsimonious migration patterns, consistent with each tree topology. Infer parsimonious migration history of metastatic tumors by MACHINA classify the patterns into 1) parallel single source seeding represents each metastatic site is seeded directly from the primary tumor, 2) single source seeding represents each metastatic site is seeded from only one other anatomical site, 3) multi-source seeding represents a metastatic site may be seeded from multiple anatomical sites without directed cycles, 4) reseeding represents metastatic site direct circles. It also provides clonality as mono- and poly-clonal patterns. The resulting solution with the lowest overall migration number and lowest comigration number were determined for each patient. No other constraints were applied to the migration plots for MACHINA.

#### Statistical analysis

The data are presented as mean ± standard deviation and compared using the Student’s *t* test or Mann–Whitney U test. For survival analysis, overall survival was defined as death from a GC diagnosis, and it was generated by Kaplan–Meier curves with a log-rank test. For multivariable analysis, the Cox proportional hazard model was used and displayed using the hazard ratio (HR) and its 95% confidence interval (CI). R version 3.6.1 (R Foundation for Statistical Computing, Vienna, Austria) was used for all statistical analyses.

## Results

### Samples of paired primary-metastatic GC

Formalin-fixed paraffin-embedded (FFPE) samples of paired primary-metastatic (including LNs) tumors were collected from 15 patients (GCM01-15) who had undergone gastrectomy and lymphadenectomy with metastasectomy (Supplementary Tables S1 and 2). Whole-exome sequencing (WES) data were available for 42 multi-regional (by invasion depth) primary tumors in 15 patients, 29 LN metastasis in 12 patients, 13 ovary metastasis (OVM) in 7 patients (single OVM was available in one patient), 9 hematogenous metastasis (8 of liver and 1 lung) in 6 patients, and 6 peritoneal metastasis in 5 patients. No patient had both hematogenous and ovarian or peritoneal metastasis simultaneously.

### Genomic architecture by metastatic routes

A total 18,567 somatic nonsynonymous variants were identified in 7854 genes in 99 samples from 15 patients (16,292 of missense, 896 of nonsense, 5 of nonstop, 373 of splice site, and 1001 of small insertion and deletion mutations). There was no evidence of microsatellite instability (MSI) or Epstein–Barr virus (EBV) in any of the tumors; therefore, the tumors were classified as chromosomal instability (CIN) or genomically stable (GS) molecular subtypes [2]. No known pathogenic germline variants were identified, including mismatch repair genes or *CDH1*, in any of the patients. We compared the somatic copy number alterations (SCNAs) and mutations between primary and metastatic tumors considering their routes. The copy number (CN) burden and ploidy were significantly higher in hematogenous metastatic tumors than in the primary tumors (*p* < 0.001, *p* = 0.0125, respectively, Fig. 1b). The median number of nonsynonymous variants per sample was 158 (range 19–824, Fig. 1c); it was higher in hematogenous and lower in peritoneal/ovarian metastatic tumors than in the primary tumors (*p* = 0.0025, *p* = 0.0034. and *p* = 0.0030, respectively, Fig. 1d). The primary tumors were classified as CIN and GS in seven and eight patients, respectively; there was no case with different subtypes within multi-regional primary tumors from an individual. In accordance with the molecular subtype of the primary tumor, an increased CN burden and number of nonsynonymous variants for the hematogenous metastatic tumors when compared to those of the primary tumors were observed in the CIN subtype (Supplementary Fig. S1A–C). In contrast, fewer nonsynonymous variants for peritoneal/ovarian metastasis without the difference in CN burden and ploidy when compared to the primary tumors was observed for the GS subtypes.

Most of the primary tumors with a CIN subtype had hematogenous metastasis, whereas the GS subtype was associated with peritoneal/ovarian metastasis. The subtypes of the metastatic tumors usually were same with the primary tumors, and molecular subtype changes were observed for three patients (Fig. 1c); subtype changes from CIN to GS were observed in peritoneal/ovarian metastasis (GCM09) and from GS to CIN in liver (GCM10) and ovarian metastasis (GCM12). The histology of the tumors supports these subtype changes as the signet ring cell and poorly cohesive carcinoma were dominant in the primary tumors (GCM10 and GCM12, respectively), while an intestinal type of glandular formation was observed in the metastatic tumors that showed molecular subtype changes from GS to CIN (Supplementary Fig. S2A, B).

To better understand the patterns of mutational accumulation in the primary and metastatic tumors by these routes, we performed mutational signature analyses of all samples. Three signature classes were mainly observed in the cohort: clock-like signatures (single-base substitution (SBS) 1 and 5), which were reported to have a high-prevalence in various cancer types, including gastric cancer [34–36] and polymerase epsilon exonuclease domain mutations (SBS10b), which are predominantly composed of C > T mutations and associated with a DNA polymerase epsilon (POLE) mutation in colorectal, endometrial, and gastric models [37]. When comparing the proportion of each signature by the route of metastasis, OVM was found to be related to lower SBS1 and SBS10b but higher SBS5 when compared with that of the primary tumors (*p* = 0.0027, *p* = 0.0071, and *p* = 0.0221, respectively, Fig. 1e). The proportion of SBS5 was lower in LN (*p* = 0.0055) but higher in hematogenous metastasis (*p* = 0.016) when compared with that in the primary tumors. As previously reported [38], a high proportion of SBS5 in peritoneal metastasis was observed; however, it was not significant.

### Genomic alterations in cancer driver genes by metastatic routes

**Fig. 2:**
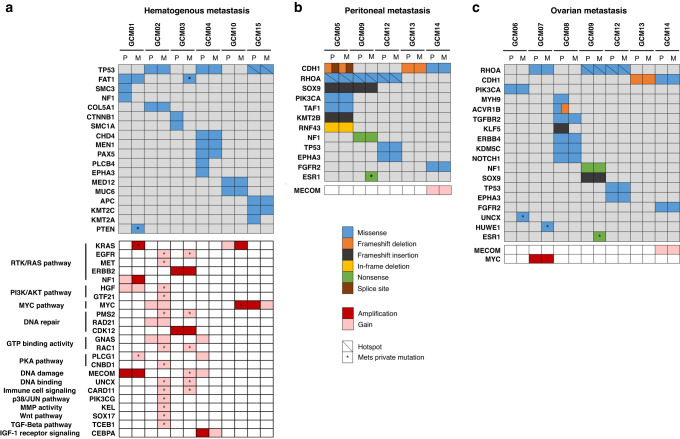
Genomic alterations of the cancer driver genes in metastatic gastric cancer by metastatic routes. Oncoprints of somatic mutations and copy number alterations are depicted for the primary and metastatic tumors of each patient with (**a**) hematogenous, (**b**) peritoneal, and (**c**) ovarian metastatic tumors. An asterisk indicates a de novo somatic mutation or gain/amplification in metastatic tumors. A diagonal line in a box indicates tumor hot spot mutations. (R175H of *TP53* mutation in GCM15; Y42C and R5W of *RHOA* mutations in GCM09, GCM12 and GCM05, respectively).

To compare the genomic alterations driving GC metastasis by their routes, we focused on the nonsynonymous mutations and gain/amplification (gain/amp) in the consensus cancer driver genes [39]. The *TP53*, *RHOA*, and *CDH1* mutations were mainly observed in hematogenous, ovarian, and peritoneal metastasis, respectively (Fig. 2a–c). As the CIN type of the primary tumor was related to hematogenous metastasis, the SCNAs of the driver genes were mainly observed in patients with hematogenous metastasis; various de novo gain/amps in the metastatic tumors were present, which activate the RTK/RAS, PI3K/AKT, and MYC signaling pathways (Fig. 2a). In contrast, a few SCNAs without the de novo gain/amp of the driver genes were observed in peritoneal/ovarian metastatic tumors. Small insertion, deletion, and nonsense mutations, which likely cause loss-of-function of proteins, were more frequently observed in peritoneal/ovarian metastasis than hematogenous metastasis (Fig. 2b, c). Despite the decreasing total number of nonsynonymous variants for peritoneal/ovarian metastatic tumors when compared to the primary tumors (Supplementary Fig. S1A–C), de novo mutations in cancer driver genes were observed in peritoneal/ovarian metastatic tumors. These findings suggest that the underlying genomic alterations driving GC metastasis may differ by routes.

### Prognosis in GC by metastatic routes and molecular subtypes

The poor prognosis of patients with diffuse histology (surrogate of GS type) of primary GC or peritoneal/ovarian metastasis when compared to intestinal histology (surrogate of CIN type) and hematogenous metastasis has been reported [40, 41]. Consequently, we further evaluated the prognosis of patients using the routes of metastasis and molecular subtypes of primary and metastatic tumors. Despite the small sample size, a trend of poor prognosis for peritoneal/ovarian metastasis and the GS type of GC was observed when compared to the hematogenous and CIN type of GC, respectively, although the difference was not significant (Supplementary Fig. S3A, B, respectively). The prognosis of the GS type of the metastatic tumors was worse than that for the CIN type (Supplementary Fig. S3C, log-rank *p* = 0.024), implying that the evolved molecular characteristics during the GC metastasis were also clinically important.

## Genetic distance among metastatic tumors

**Fig. 3:**
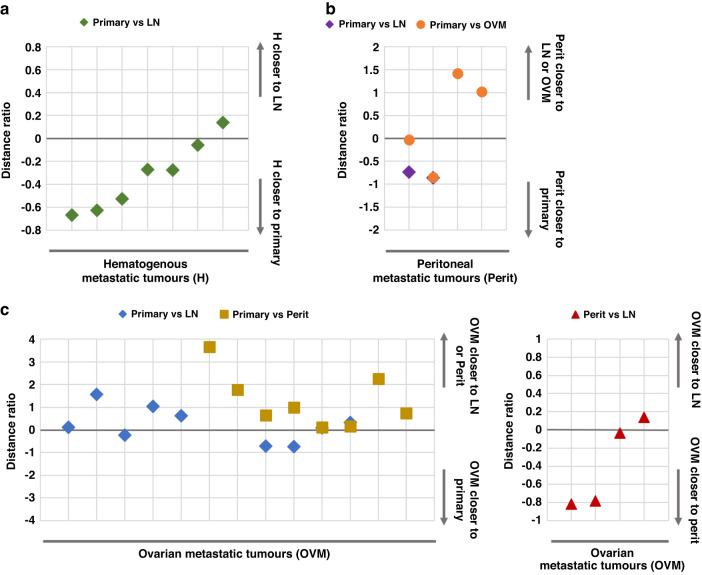
Genomic distances among the primary and metastatic tumors based on their routes in the paired samples. The distance ratio was calculated as d(A to B)/d(A to C)-1. A distance ratio > and < 0 indicates that A is genomically closer to C and B, respectively. **a** Distance ratio plot showing that hematogenous metastasis (H) was closer to the primary tumor when compared to lymph node (LN) metastasis. **b** The genomic distance of peritoneal metastasis (perit) when compared to LN and ovarian metastasis (OVM) showed peritoneal metastasis to be closer to the primary tumor than LN metastasis. **c** Genomic distance analysis of the OVM to the primary, LN, and peritoneal metastasis showed that OVM is genomically closer to LN or peritoneal metastasis when compared to the primary tumor (left), and closer to the peritoneal when compared to LN metastasis (right).

To elucidate the origin of the metastatic tumors via their routes, we calculated the genomic distance among primary and metastatic tumors in each patient. The genomic distance of hematogenous metastasis was closer to the primary tumor than LN metastasis. (Fig. 3a) This indicates that the hematogenous metastasis of GC originated directly from primary tumors through blood vessels and fundamentally different progress mechanisms to LN metastasis, as with other cancer types [30, 42–44]. Peritoneal metastasis was also closer to the primary tumor than LNs metastasis (Fig. 3b), implying that it occurs directly from the primary tumor rather than LNs; however, this result was derived from only two samples. In the case of OVM, its genomic distance was closer to the LN/peritoneal metastasis than to the primary tumors (Fig. 3c). Furthermore, OVM was closer to the peritoneal metastasis than to LN metastasis. Comparing the genomic distance of each metastatic tumor to the invasion depth of the primary tumor (tumors in inner [Ti] and outer [To] layer), the metastatic tumors were usually found to be closer to To than to Ti (Supplementary Fig. S4A–C), suggesting that GC metastasis occurs while cancer cells penetrate the outer layer of stomach rather than during the early stage of cancer in the mucosa layer. Considering the tier system for metastatic LNs, both the genomic distance of the D1 (perigastric) and D2/D4 (extra-perigastric/distant) LNs were found to be closer to To than to Ti without any definite difference (Supplementary Fig. S4D).

### Inferring the phylogeny and migration patterns of metastatic GC

**Fig. 4:**
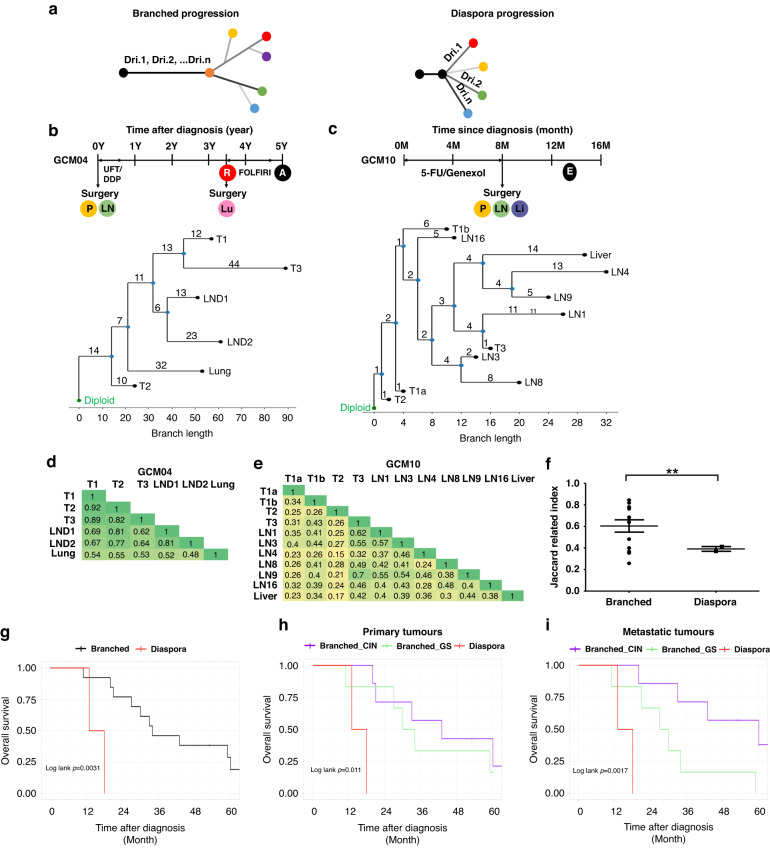
Inferred phylogeny and migration patterns of metastatic gastric cancer with genomic similarity and the resulting prognosis. **a** Conceptual figures depicting two distinctive metastatic progression patterns, branched and diaspora. Clinical course and phylogenetic tree (by MEDICC2) of a representative case of (**b**) branched progression (GCM04) and (**c**) diaspora progression (GCM10). **d**, **e** Heatmap of pairwise Jaccard indices, representing genomic similarity between tumors from an individual, in each representative case of branched (GCM04) and diaspora (GCM10) progression. **f** Statistical comparison of the average Jaccard index between patients with branched versus diaspora progression. Diaspora progression showed a lower Jaccard index, implying more genetic inter-tumoral heterogeneity when compared to branched progression (**; *p* < 0.005). Kaplan–Meier’s curves for overall survival (OS) based on (**g**) metastatic migration patterns and migration patterns with the molecular subtypes of (**h**) primary tumor and (**i**) metastatic tumors. The diaspora progression was related to poor prognosis when compared to branched progression (*P* = 0.0031). When considering the molecular subtypes of primary or metastatic tumors together, both the migration pattern and subtype of the metastatic tumors were found to be related to the patients’ survival (*p* = 0.0017, **i**). P primary tumor, LN lymph node, Lu lung metastasis, R recurrence, A alive, Li liver metastasis, E expired.

To evaluate the evolutionary progression of metastatic GC, the phylogeny of the tumors was inferred for each patient. We conducted both mutation- (Treeomics) and SCNA- (MEDICC2) based phylogeny analysis and found that the phylogenic trees of each patient were consistent (Supplementary Figs. S5 and 6, Supplementary Table S5). The phylogenic trees could be classified into two distinct migration patterns: branched and diaspora progression (Fig. 4a). The branched progression shared a long trunk that harbored the initial cancer driver alteration, indicating that a founder clone that acquired driver alteration disseminated late from the primary tumor and evolved into the metastatic tumors. (Fig. 4b) The diaspora progression shared a short trunk, and the phylogenetic tree involved multiple branches from a founder clone. Each metastatic tumor was found to have distinct driver alteration, indicating that the metastatic subclones had not yet acquired the driver gene alterations disseminated from the primary tumors, and that they evolved independently of each other, resulting in substantial genetic divergence between the primary and metastatic tumors. (Fig. 4c) [30, 31]

The diaspora progression is expected to be related to the high intertumoral genetic heterogeneity and consequent poor prognosis; [30, 45] thus, we analyzed the genetic similarities of the tumors in each patient. We found that the Jaccard index among the tumors, representing genetic similarities, was high in branched progression but low in diaspora progression (Fig. 4d–f, *p* = 0.0044, Supplementary Fig. S7A). This suggests that the diaspora pattern is related to high intertumoral heterogeneity in metastatic GCs. The different phylogenetic patterns and genetic similarities of the metastatic GC could be related to the clinical outcomes; thus, we conducted survival analysis using the migration pattern. A trend of poor prognosis in patients with low genetic similarities (high intertumoral heterogeneity) was observed, though it was not significant (Supplementary Fig. S7B, *p* = 0.09). The prognosis of patients with the diaspora progression for phylogenetic metastasis was poorer than that of patients with branched progression (Fig. 4g, log-rank *p* = 0.0031). Considering the molecular subtypes of the primary and metastatic tumors of the patients together, diaspora progression showed the worst prognosis and branched progression differed in accordance with the molecular subtypes of the metastatic tumors but not the primary tumors (Fig. 4h, i), indicating the phylogeny of GC metastasis to be related to the patients’ prognosis.

Next, we inferred the migration history of metastatic GC in each patient to understand their clonal origins. The metastatic tumors of GC were generally found to be of polyclonal origin, and the inferred parsimonious migration history was single source seeding in most cases (Supplementary Table S5 and Supplementary Fig. S8). Considering the multiregional samples of the primary tumor, all 15 cases were estimated as parallel single source seeding, and no multisource or reseeding was estimated, indicating that most GC metastasis was from the primary tumor both directly and independently.

### Estimation of the evolutionary history of the LN metastasis of GC

**Fig. 5:**
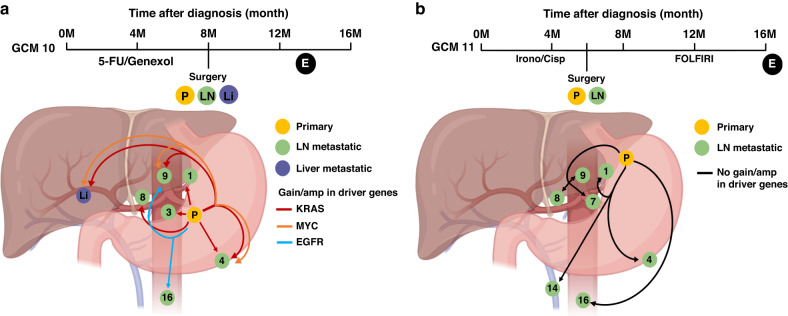
Two representative cases showing the estimated evolutionary history of lymph node metastasis in gastric cancer considering their anatomical locations based on the inferred phylogenies and migration history. **a** In patient GCM10, the primary tumor was located in the mid-body of the stomach and there was metastasis to multiple lymph nodes (LNs) including para-aortic LN (#16), a distant LN, and the liver. **b** In patient GCM11, the primary tumor was located in the upper body of the stomach and metastasis for multiple LNs was observed, including in superior mesenteric vein (#14v) and for para-aortic LN (#16); both were regarded as distant LNs. In both cases, the inferred migration history of the LN metastasis was polyclonal parallel single source seeding, indicating that each LN metastasis was from the primary tumor directly rather than sequential metastasis between the proximal to distal tumors. P primary tumor, LN lymph node, Li liver metastasis, E expired.

Based on the inferred phylogeny and migration history of the metastatic tumors, we estimated the evolutionary history of LN metastasis for GC considering its anatomical locations. To do this, we assumed the following: 1) as the lymphatic flow is unidirectional, LN metastasis follows a proximal to distal pattern; 2) clonal sweep or vanished clones by immune clearance are not considered [46]. Two representative cases of migration history in patients with multiple LN metastasis including distant LNs (D4 level) are shown in Fig. 5. The patient with GCM10 had LN metastasis at #1, 3, 4 (D1 level), 8, 9 (D2 level), 16 (D4 level), and liver metastasis and all samples were exposed to preoperative chemotherapy and retrieved simultaneously (Fig. 5a). The molecular subtypes of the primary and most of the metastatic tumors were GS, but it was annotated as CIN in the tumor of LN #4, 9, and liver metastasis. (Fig. 1c) The primary tumor harbored *KRAS* gain/amp, and it was sustained in all metastatic tumors except #16 LN, a distant LN. (Supplementary Fig. S9A) There were de novo *MYC* gain/amps in LN #4, 9, and liver metastasis, and de novo *EGFR* gain/amps in LN #9 and 16, respectively. Considering the phylogenetic trees and inferred migration history of LN metastasis (Supplementary Figs. S5–6, 8, and 9B), each metastatic tumor was found to have directly originated from the primary tumor (Fig. 5a). The GCM11 patient had LN metastasis at #1, 4, 7 (D1), 8, 9 (D2), 14v, and 16 (D4). All tumors were annotated as CIN subtypes, and no gain/amp was observed in the cancer driver genes (Figs. 1c and 5b). All primary and metastatic tumors were obtained at the same time after chemotherapy. The inferred migration history of LN metastasis for this patient (Supplementary Fig. S5–6, 8, and 9C) also showed that each metastatic LN originated from the primary tumor. These findings suggest that each LN metastasis of GC is an independent event rather than a sequential one from D1 to D2/D4.

## Discussion

Like Paget’s *“seed and soil”* hypothesis, the common metastatic routes and organs differ in accordance with the site of the primary cancer [47]. The different correlations for chromosomal instability and tumor burden in metastatic tumors by primary cancer types and the genomic landscape of metastasis differed according to the reported target organs [3, 48, 49]. Chromosomal instability and mutations promotes tumor progression by increasing subclonal diversity and tumor evolution [50, 51]. Our analysis of genomic alterations from paired primary and metastatic GC samples based on their route showed increased chromosomal instability, including the presence of de novo gain/amp in driver genes only in hematogenous metastasis, while this was not found in ovarian/peritoneal metastasis; instead, de novo somatic mutations were mainly observed. These findings imply that the genomic alterations driving cancer metastasis differ not only by the primary tumor itself but also by the metastatic routes. Cancer cells from the primary tumors of GC naturally invade the serosa, the outer layer of the stomach wall, and are exposed to the peritoneal space and cause peritoneal metastasis [52]. The peritoneal cavity is a sterile environment, and prior to the patient experiencing peritonitis or cancer, it is rarely exposed to invaders; [53] consequently, immune surveillance of this space will be different to that of blood where immune cells are highly enriched. In this sense, the peritoneal space may be a less harsh environment for cancer cells when compared to blood, and therefore, less aggressive genomic changes could lead to peritoneal metastasis instead of hematogenous metastasis. However, further investigations are required to validate this hypothesis.

The phylogeny represents the evolution of cancer metastasis at the genomic level. Various phylogeny patterns (branched, linear, and diaspora) have been reported in multiple cancer types. Among them, metastatic cancer through diaspora progression, in which metastatic tumors evolve early and independently of each other, has high intertumoral heterogeneity [30, 31]. Similar to intratumoral heterogeneity, high intertumoral heterogeneity among metastatic tumors indicates chemotherapy resistance and treatment failure, which are main reasons for poor prognosis [45, 54, 55]. The results of the present study showed that patients with diaspora progression of GC metastasis were related by their high intertumoral heterogeneity (low similarity) with poor prognosis. This suggests that phylogeny and intertumoral heterogeneity are clinically important as positive indicators of the patients’ prognosis. Additionally, the diverse molecular characteristics of cancer cells vary in their sensitivities to chemotherapy; therefore, chemotherapy with combination of drugs [56] may be more effective than a mono-drug regimen [57] to the patients with diaspora pattern of metastasis. However, standardized methodology to define phylogeny patterns and intertumoral heterogeneity should be developed and validated via clinical studies.

As metastatic tumors ultimately cause the patients’ death, stronger positive association of the metastatic tumor subtypes than that of the primary tumor to the patients’ prognosis found in this study is understandable. This indicates that identifying the molecular characteristics of not only primary but also the metastatic tumors could be clinically important for enabling precision oncology. In addition, de novo SCNAs in cancer driver genes in hematogenous metastatic tumors indicate that the concept of targeted treatment could be expanded to include potential molecular drivers (i.e., NCI-MATCH, National Cancer Institute Molecular Analysis for Therapy Choice, trial) [58] of metastatic tumors. For example, de novo *MYC* and *EGFR* gain/amp in liver and distant LN (#16) metastasis were observed in GCM10; as the efficacy of targeting each of *MYC* and *EGFR* has been reported [59–61], therefore, the strategy targeting both *MYC* and *EGFR* simutaneously focusing on the metastatic tumors might be a potential treatment option for the patient. In the ovarian/peritoneal metastatic tumors, some de novo somatic mutations were observed in the cancer driver genes. A recent study demonstrated a significant correlation between *TP53* and *MADCAM1* mutations and poor metastasis-free survival of gastric cancer. Furthermore, the study showed that *MADCAM1* mutation promotes cancer cell migration and triggers tumor metastasis by establishing an immune-suppressive microenvironment [62]. In the present study, we did not identify any mutations in the *MADCAM1* gene in either the primary or metastatic tumors. The low number of patients in this study may not have been sufficient to detect rare mutation, such as those observed in 1–3% of primary gastric cancer cases [2, 63].

The OVM, also called the Krukenberg tumor, is a unique pattern of cancer metastasis that occurs in women. It originates mainly from GC but also from various primary tumors including colorectal and breast cancer [7, 64]. The mechanism of how extra-ovarian tumors cause ovarian metastasis remains unclear; however, the frequent association of lymphovascular invasion with LN metastasis and the tumor involves the cortex of ovary rather than its surface, implying that OVM is a kind of lymphatic spread. Signet ring cell carcinoma, a main cell type of OVM, is a well-known cause of peritoneal metastasis that is a common concomitant metastasis to OVM in GC, suggesting it is a phenotype of peritoneal metastasis. The present results showed that the genomic distance of OVM was close to that of peritoneal metastasis, while the migration history showed that the OVM was from the primary tumor directly rather than via peritoneal or LN metastasis. In another study, GC patients with only OVM had better prognosis when compared to patients with other types of metastases [65]. Therefore, OVM could have a pattern of peritoneal metastasis or an independent and distinctive metastatic pattern. Furthermore, the OVM of breast cancer seemed to be a hematogenous metastasis as chances of direct exposure of breast cancer cells to ovary are nil; [64] this indicates that the mechanism of OVM could differ from that of primary tumor. Further genomic studies focusing on OVM from various primary cancer types will provide more clues to better understand its mechanisms.

LN metastasis is the most common pattern of GC metastasis and LN status is one of the most pivotal clinical factors related to patient prognosis and used as a staging system [19]. As the lymphatic network around the stomach is complicated, predicting the presence and location of the LN metastasis of GC is difficult; [8] thus, guidelines recommend radical surgery and lymphadenectomy at the D1 and D2 level [66]. Traditionally the LNs around the stomach are labeled based on their anatomical location and grouped as a tiered system as follows: D1 (perigastric), D2 (extra-perigastric), and D4 (distant) [10]. As it is assumed that LN metastasis occurs from proximal to distal, LN metastasis of the GC is thought to be sequential, from D1 to D2/D4 in the clinic [11]. However, there is a clinical observation called “skip metastasis” indicate the presence of metastatic LNs in an extra-perigastric (D2 level) without perigastric (D1) involvement [67]. In our analysis, each LN metastasis, even for distant LNs (#14v, 16), was inferred as having parallel single source origins from a primary tumor directly rather than from subsequent linear metastasis from other metastatic LNs closer to the primary tumor. In addition, no difference in the genomic distance between metastatic LNs at the D1 and D2 levels in relation to the layer of the primary tumor was found. These findings support the phenomenon of skip metastasis and that the anatomy-based LN tiered system of GC is not genomically different.

This study had certain limitations. First, our samples were surgically resected specimens, and patients with metastatic tumors with no prior surgical indication were not considered; therefore, the findings may not cover inoperable metastases. Although the primary tumors were dissected by the layer of gastric wall, small number of samples (1–4) for each primary tumor may not be enough to cover intratumoral heterogeneity of the primary tumor [13, 14]. A small number of cases, especially considering the routes of metastasis, may not be enough to cover all possible patterns of phylogeny for metastatic GC [31, 68, 69]. Some samples were exposed to chemotherapy, but we did not consider the clonal selection and selective pressure from chemotherapy [70]. Further, some metastatic samples were from secondary operations or later, after surgery for the primary tumor, which may have affected the results.

**Fig. 6:**
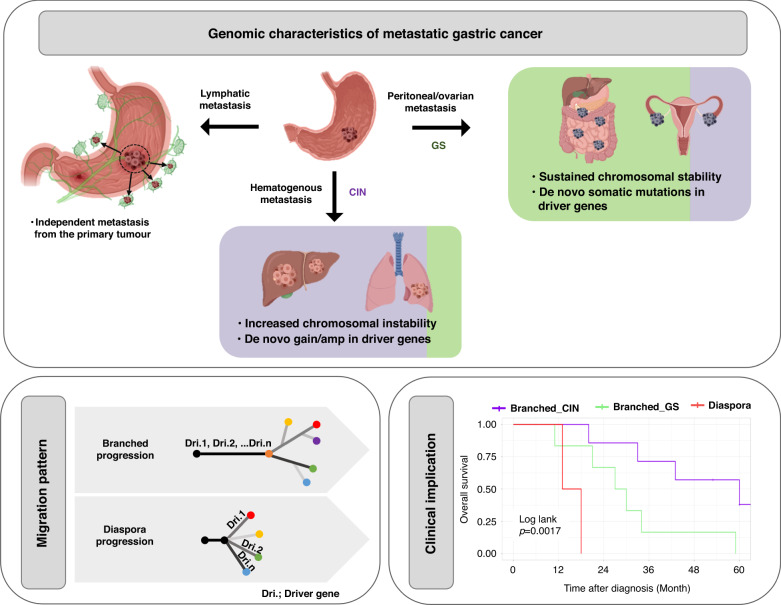
The genomic and evolutionary characteristics of metastatic gastric cancer. Comparisons between the genomic characteristics of the paired primary and metastatic gastric cancers showed the following: 1) Each LN metastasis is an independent event regardless of the anatomical location-based tier system; 2) Molecular subtype changes from chromosomal instability (CIN, purple) to genomic stable (GS, green) in 1 (peritoneal/ovarian metastasis) out of 7 cases, and GS (green) to CIN (purple) in 2 (liver and ovarian metastasis) out of 8 cases were observed between the primary and metastatic tumors, respectively; 3)The CIN subtype of the primary tumor is usually associated with hematogenous metastasis which increases the copy number burden including de novo gain/amp in cancer driver genes; 4) The GS subtype is commonly associated with peritoneal/ovarian metastasis which is sustained chromosomal stability with de novo somatic mutation in cancer drivers. Phylogeny analysis identified two types of migration patterns, branched and diaspora progression. Both molecular subtypes of the metastatic tumors and the migration patterns are associated with patient survival.

The genomic characteristics of metastatic gastric cancer showed that each LN metastasis is an independent event regardless of the anatomical location-based tier system (Fig. 6). The CIN subtype of the primary tumor is usually associated with hematogenous metastasis which increase the CN burden, including de novo gain/amp in cancer driver genes. On the other hand, the GS subtype is commonly associated with peritoneal/ovarian metastasis which is sustained chromosomal stability with de novo somatic mutations in cancer drivers, suggesting that cancer driven alterations differ in their metastasis routes from GC. The molecular subtypes of the metastatic tumors are associated with the patient’s prognosis, and metastatic tumor specific targetable alterations could have potential clinical implications on disease management. Two types of migration patterns were observed in the metastasis of GC: branched and diaspora. Both molecular subtypes of metastatic tumors and migration patterns are associated with patient survival, indicating the necessity for the genomic evaluation of metastatic GC tumors.

## Supplementary information

Supplementary results.

## Data Availability

Raw data for the whole-exome sequencing (WES) reported in this manuscript have been deposited into a sequence read archive (SRA) database with BioProject accession number PRJNA836285. For more detailed information, please contact the corresponding author (YYC).

## Code availability

The code used for the analysis is available upon request. For more detailed information, please contact the corresponding author (YYC).
