# Supplementary material for: Genomic and evolutionary characteristics of metastatic gastric cancer by routes
Source: Br J Cancer. 2023 Jul 8;129(4):672–82. doi: 10.1038/s41416-023-02338-3 (PMC10421927; doi:10.1038/s41416-023-02338-3)
Supplement: Supplementary file 1 — Supplementary results. [file 41416_2023_2338_MOESM1_ESM.docx]

Supplementary Materials for

**Genomic and evolutionary characteristics of metastatic gastric cancer by routes**

Jae Eun Lee, Ph.D.^1,2↟^, Ki Tae Kim, Ph.D.^3↟^, Su-Jin Shin, M.D., Ph.D.^4^, Jae-Ho Cheong, M.D., Ph.D.^2*^, and Yoon Young Choi, M.D., Ph.D.^5*^,

*Correspondence:

Jae-Ho Cheong, M.D., Ph.D.

Department of Surgery, Yonsei University College of Medicine,

50 Yonsei-ro, Seodaemun-gu, 120-752, Seoul, Korea

Tel: +82-2-2228-2094; Fax: +82-2-313-8289; E-mail: JHCHEONG@yuhs.ac

Yoon Young Choi, M.D., Ph.D.

Department of Surgery, Soonchunhyang Bucheon Hospital, Soonchunhyang University College of Medicine, 170 Jomaru-ro, Wonmi-gu, Bucheon-si, Gyeonggi-do, Republic of Korea

Tel: 031-782-8653, Zip: 14584

Email: [laki98@naver.com](mailto:laki98@naver.com)

**This PDF file includes:**

Figure S1.

Figure S2.

Figure S3.

Figure S4.

Figure S5.

Figure S6.

Figure S7.

Figure S8.

Figure S9.

Table S1.

Table S2.

Table S3.

Table S4.

Table S5.


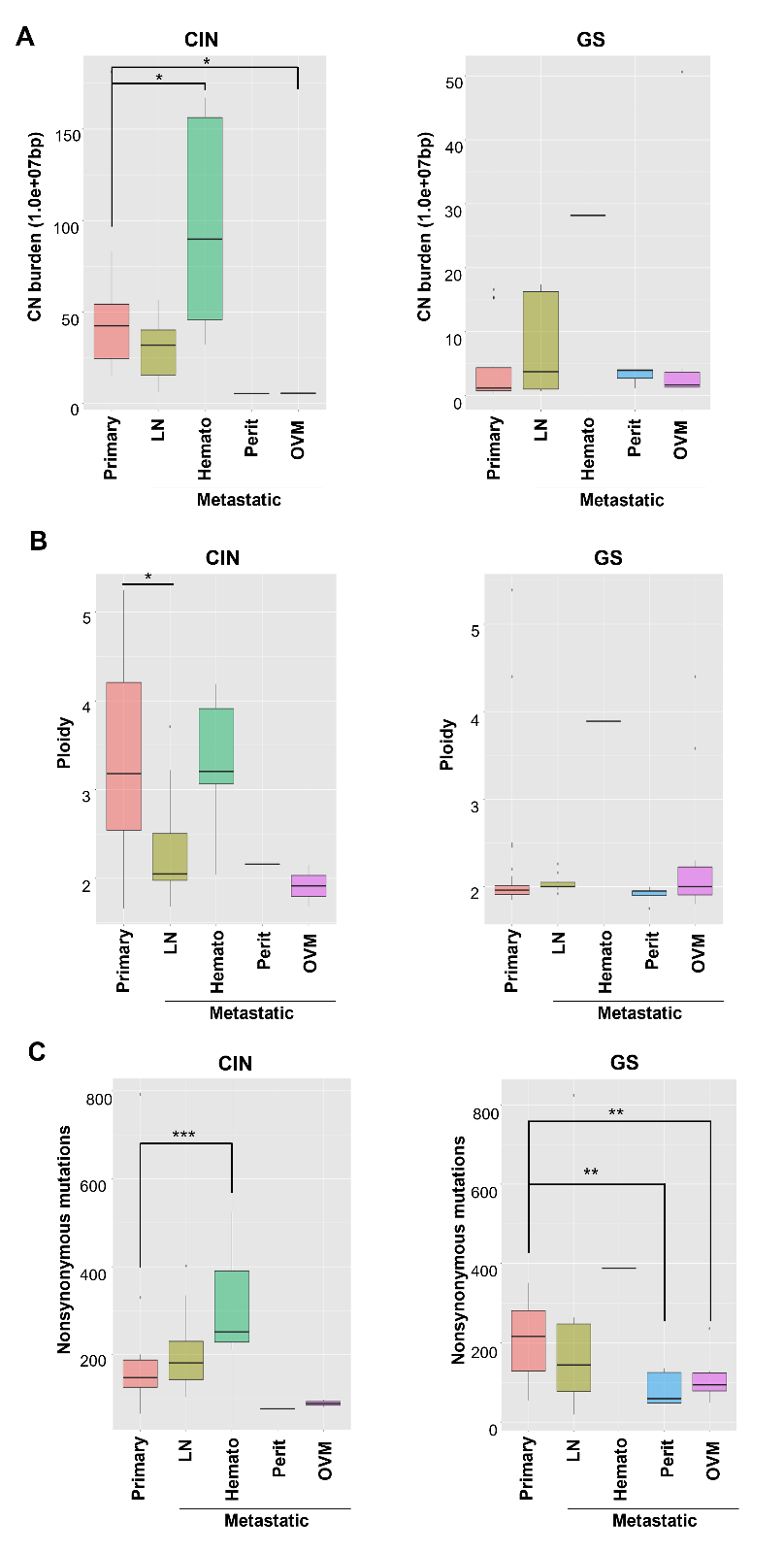


**Figure S1. Comparison of the genomic characteristics of gastric cancer.** (A) Copy number burden; (B) ploidy; (C) number of nonsynonymous mutations in primary and metastatic gastric cancers by the chromosomal instability (CIN) and genomic stable (GS) molecular subtypes of primary gastric cancer. *, **, and ***, indicate P < 0.05, < 0.005 and < 0.001 using the Mann–Whitney test, respectively. LN; lymph node, Hemato; hematogenous metastasis, Perit; peritoneal metastasis, OVM; ovarian metastasis


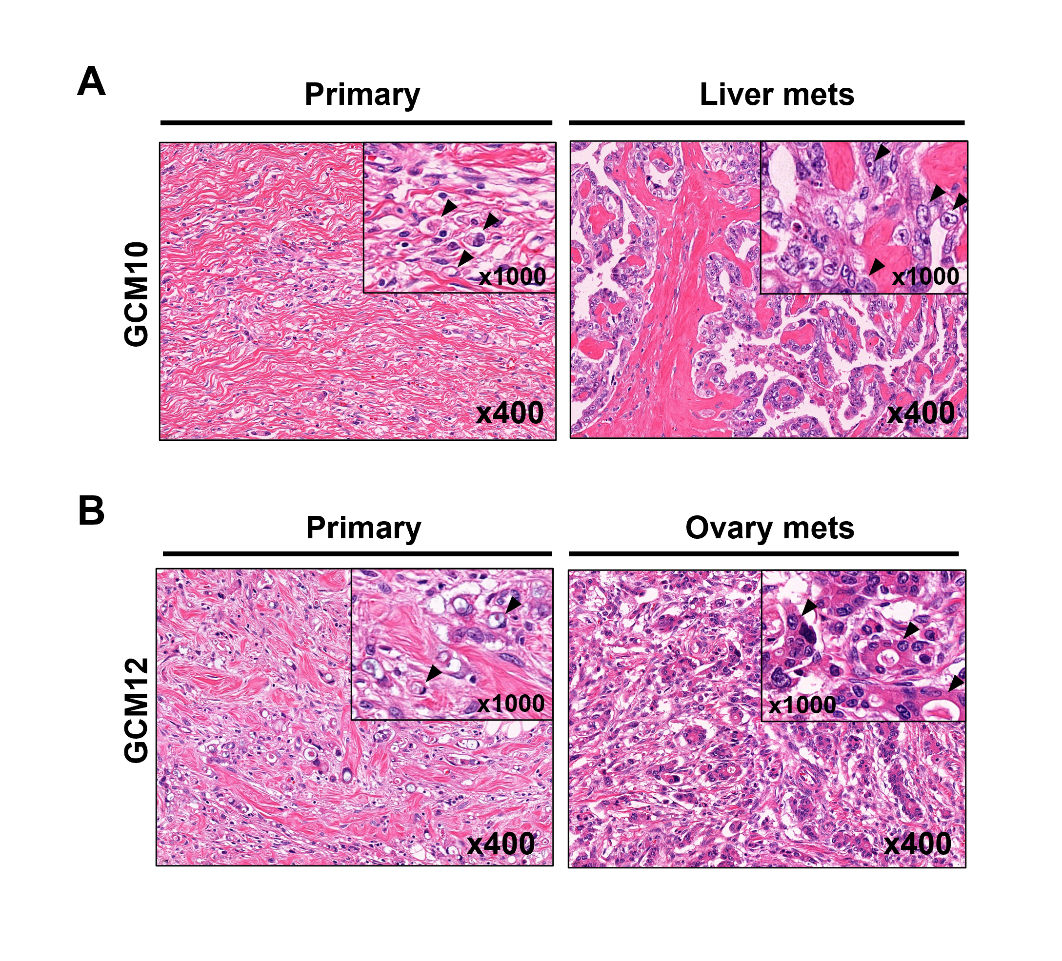


**Figure S2. Representative histology for cases with molecular subtype changes between the primary and metastatic tumors.** (A) In GCM10, signet ring cells are predominant in the primary tumor (> 90%) while intestinal type cells were mainly observed (90%) in liver metastasis. (B) In GCM12, poorly cohesive carcinoma with signet ring cells are observed in the primary tumor overall, while gland formation was observed with ovarian metastasis. For both cases, the molecular subtype of the primary tumor was genomic stable but chromosomal instability in metastatic tumors was observed.


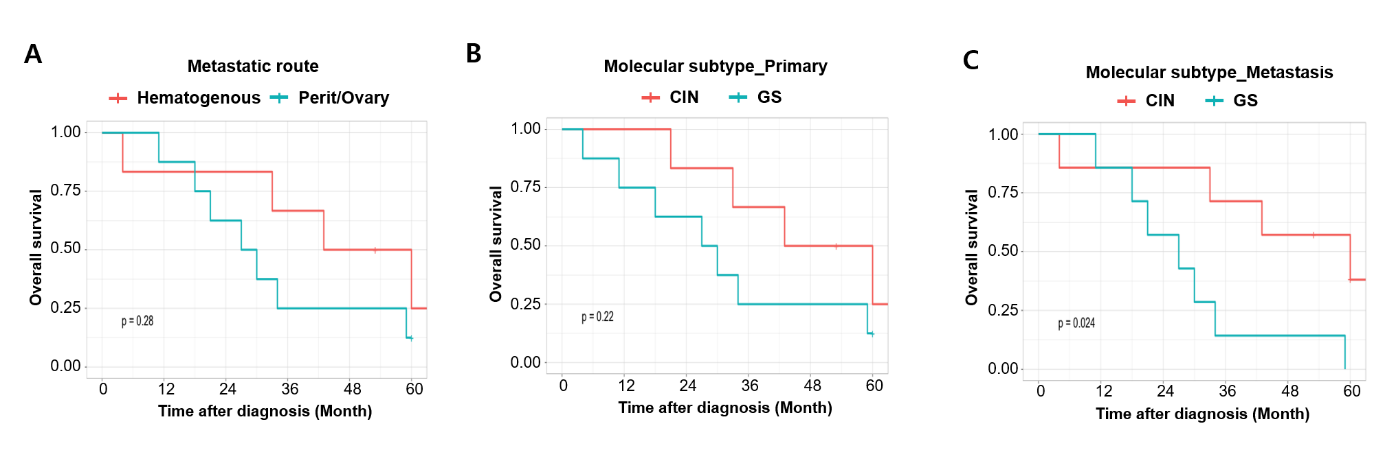


**Figure S3. Kaplan–Meier curves for the overall survival of patients with metastatic gastric cancer in accordance with the metastatic routes and molecular subtypes.** (A) Comparison of the survival of patients with hamatogenous and peritoneal or ovarian (Perit/Ovary) metastasis. Comparison of the survival of patients with chromosomal instability (CIN) and genomic stable (GS) subtypes of (B) primary tumors and (C) metastatic tumors.


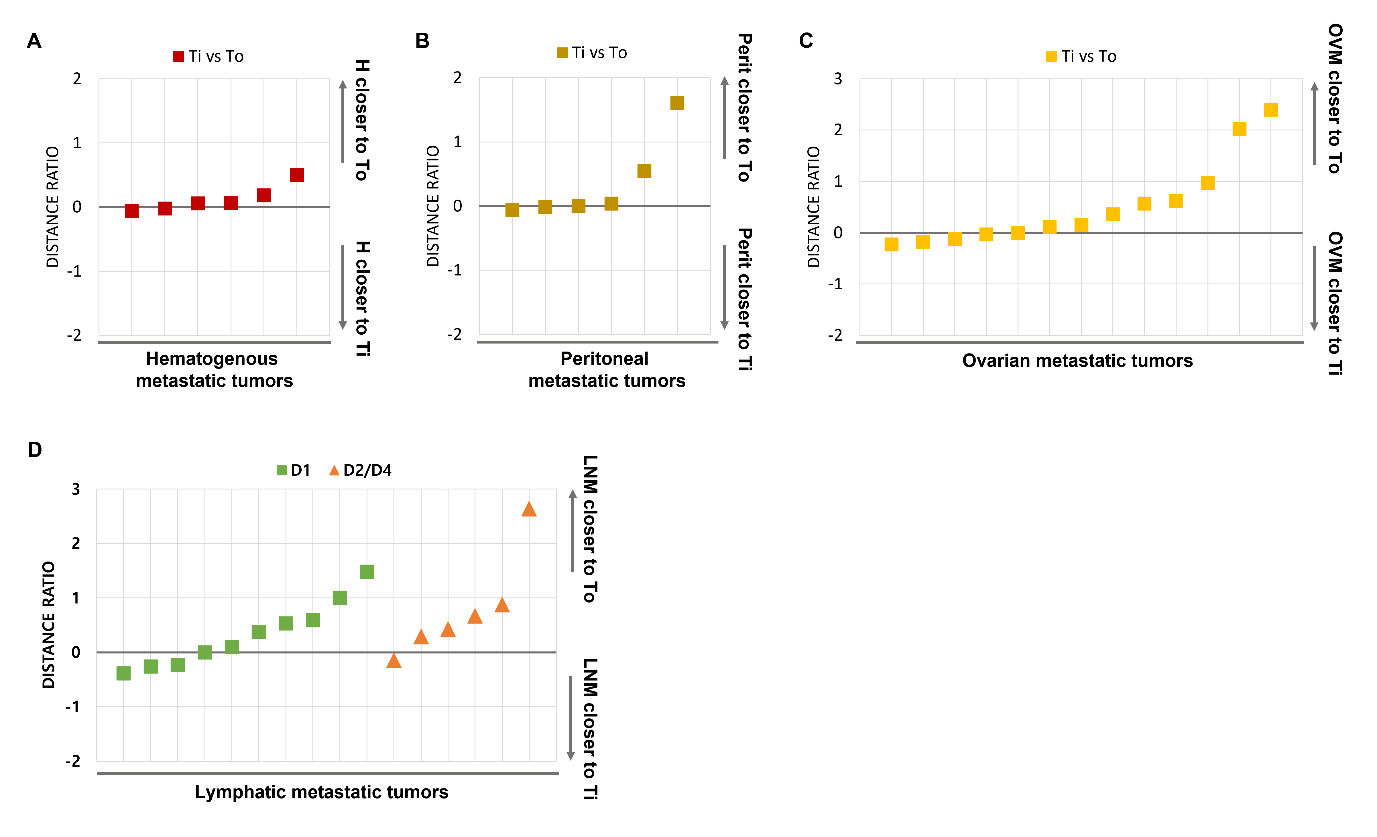


**Figure S4. Genomic distance of metastatic tumors in relation to the layer of primary tumors by route.** The distance ratio was calculated as d(A to B)/d(A to C)-1. A distance ratio >and < 0 indicates that A is genomically closer to C and B, respectively. The genomic distance of the metastatic tumors, (A) hematogenous, (B) peritoneal, and (C) ovarian metastasis were all found to be closer to the primary tumors at the outer layer (To) when compared to the inner layer (Ti). The genomic distances of the metastatic lymph nodes at D1 (perigastric) and D2/D4 (extra-perigastric/distant level) were closer to To than to Ti.


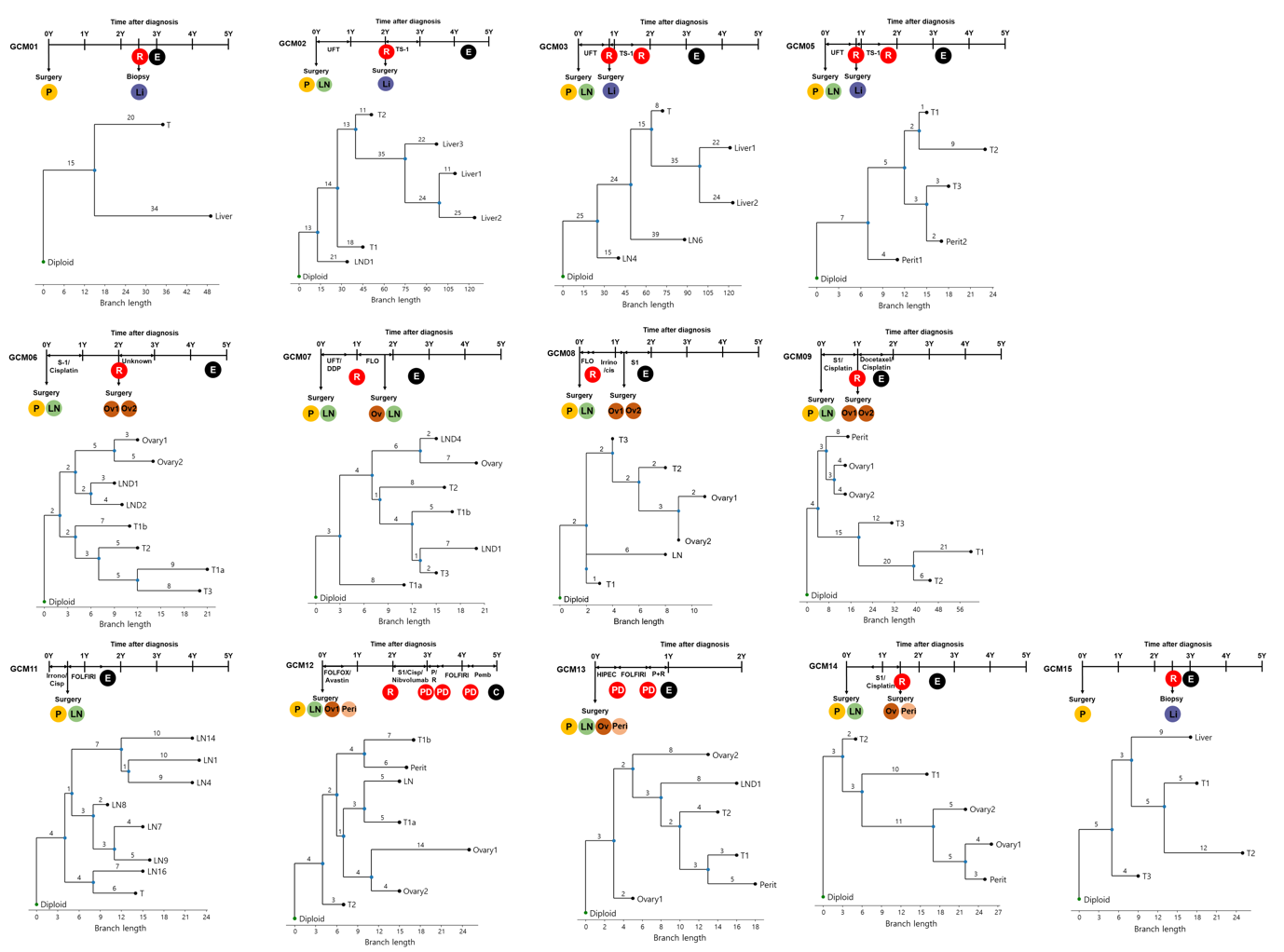


**Figure S5. Clinical course for each patient with metastatic gastric cancer and inferred phylogeny by MEDDIC2, a somatic copy-number based algorithm.** The results of GCM04 and GCM10 are presented in Figure 4 B and C, respectively, and not included in this figure.


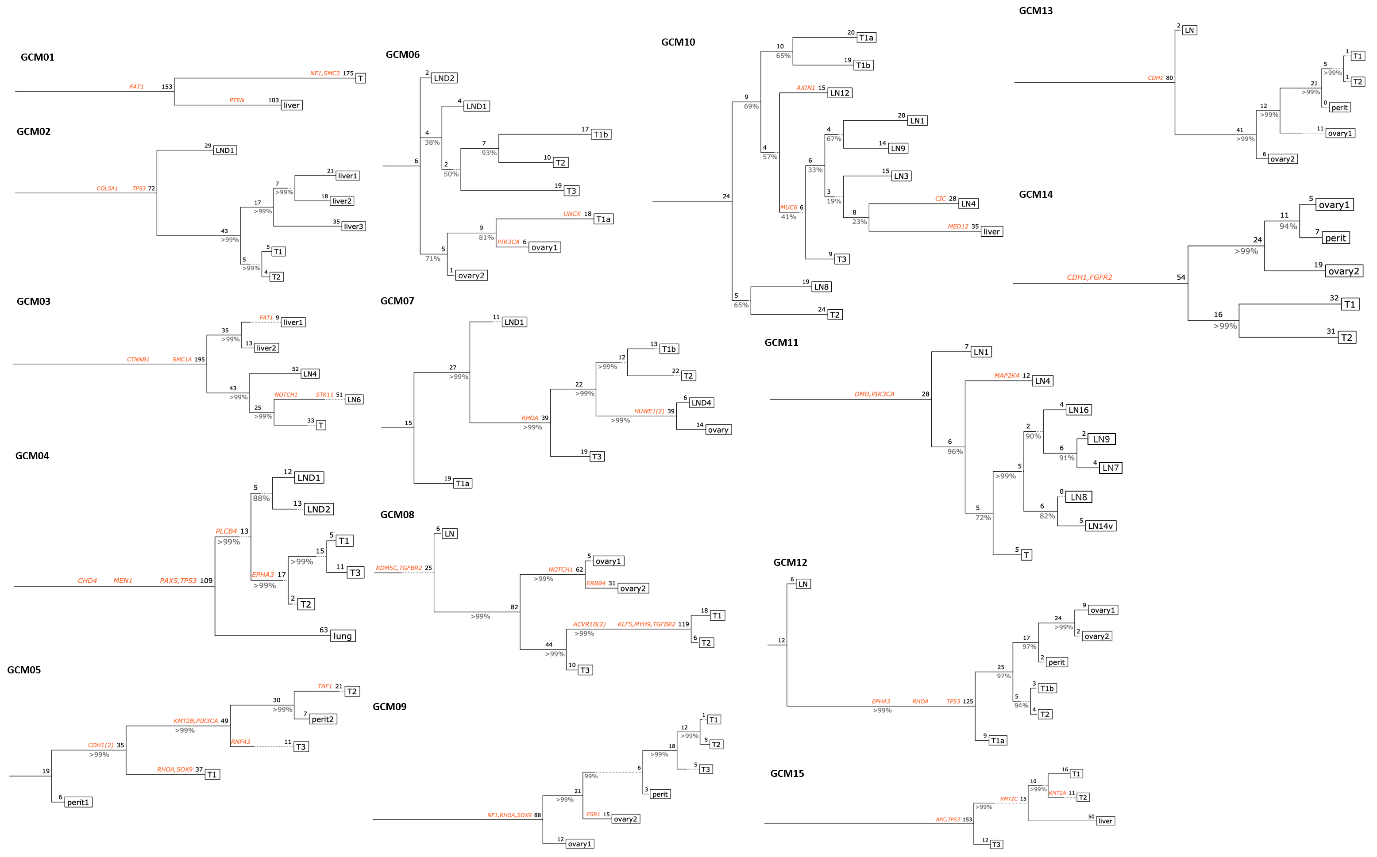


**Figure S6. Inferred phylogenetic trees by Treeomics, a mutation-based algorithm, for each patient with metastatic gastric cancer.**

**
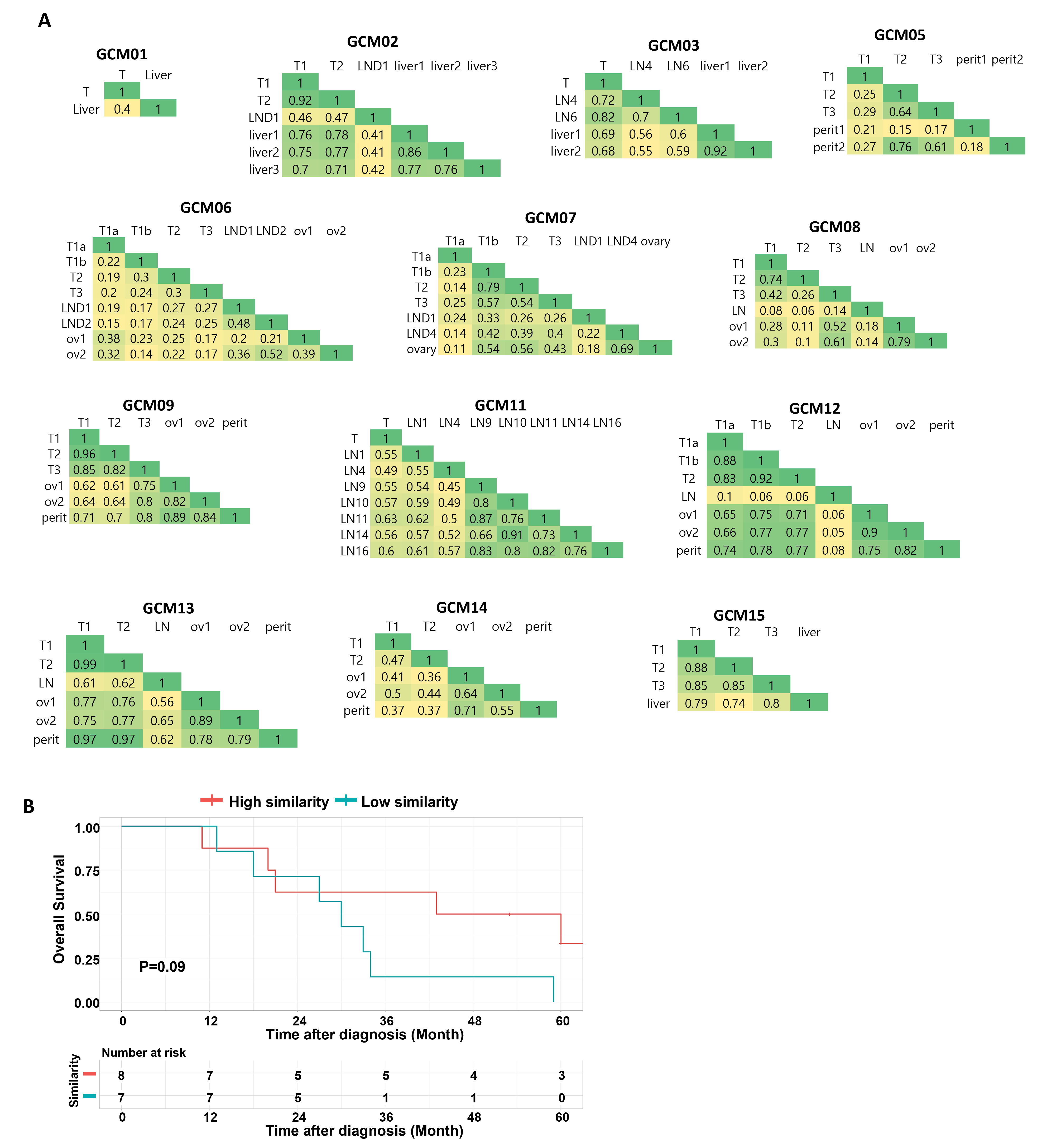
**

**Figure S7. Genetic similarity between the tumors of each individual patient with metastatic gastric cancer and its prognosis.** (A) Heatmap showing the pairwise Jaccard indices, representing genetic similarity between the tumors of each individual patient with metastatic gastric cancer, (B) the patients’ prognosis by genetic similarity (grouped by median value).

**
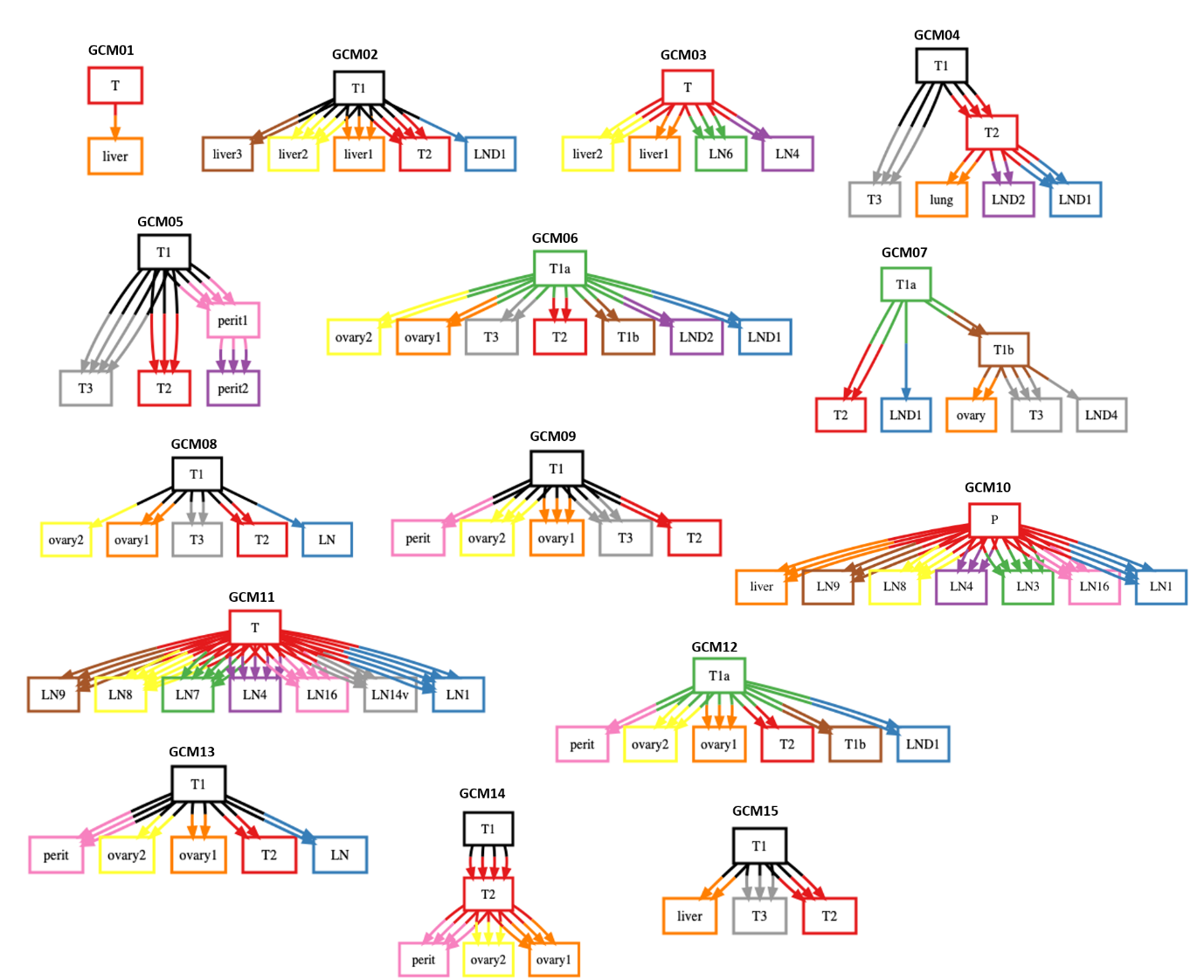
**

**Figure S8. Inferred migration history of the metastatic tumors as determined by MACHINA in each patient with metastatic gastric cancer.**

**A**


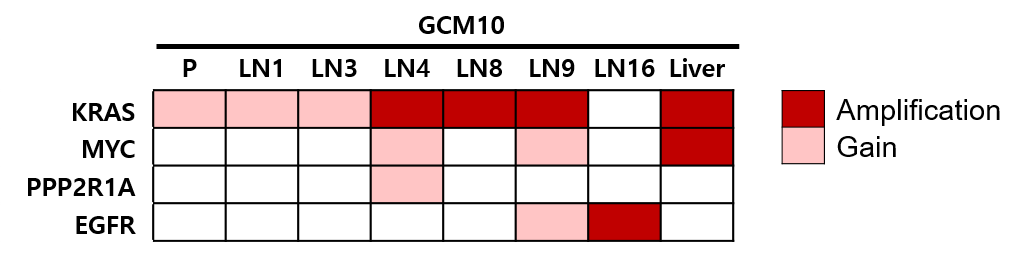


**B**


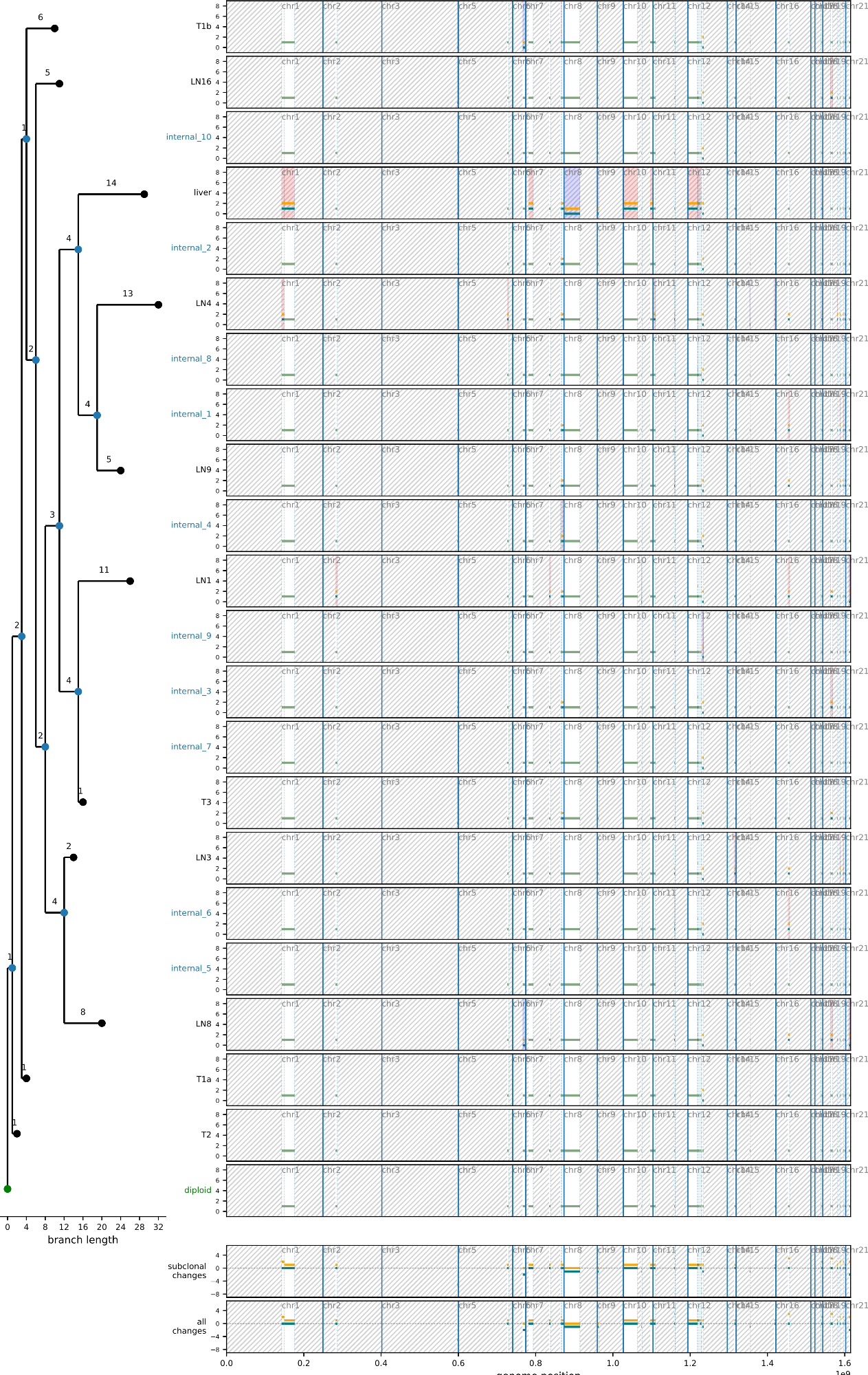


**C**

**
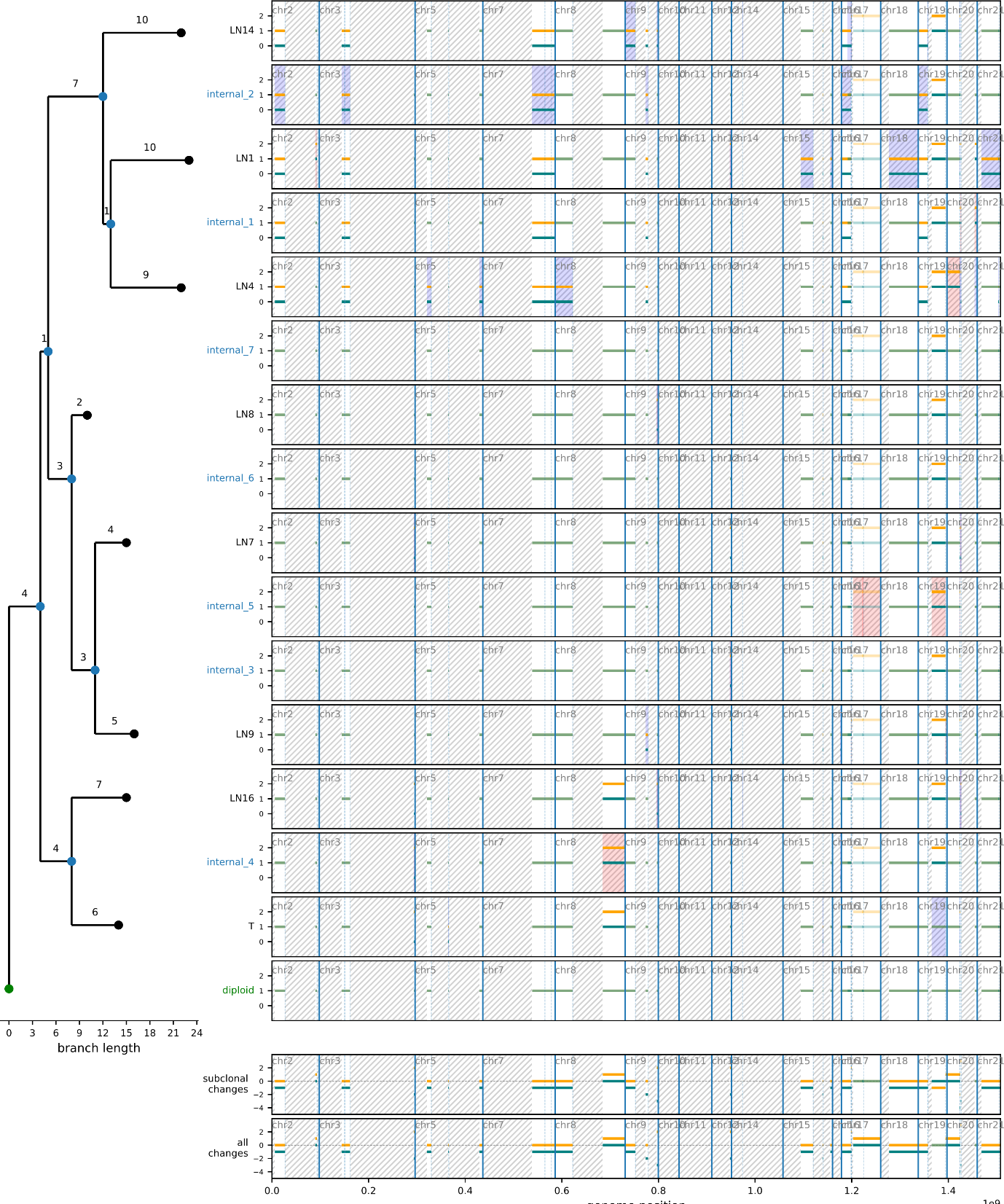
**

**Figure S9. Detailed somatic copy number changes in two representative cases (GCM10 and GCM11) of patients with multiple and distant lymph node metastasis.** (A) Heatmap showing the gain/amplification of cancer driver genes in GCM10. The phylogeny trees and subclonal changes that were inferred by MEDICC2 in GCM10 (B) and GCM11 (C).

**Table S1. Pathological information for the gastric cancer patients enrolled in the study**

| **PatientID** | **Age** | **Sex** | **TNM stage** | **Lauren** | **Histology** |
| --- | --- | --- | --- | --- | --- |
| GCM01 | 68 | M | IA | Intestinal | MD |
| GCM02 | 73 | M | IIB | Intestinal | MD |
| GCM03 | 55 | M | IIB | Intestinal | MD |
| GCM04 | 43 | M | IIIB | Intestinal | MD |
| GCM05 | 47 | F | IIIA | Mixed | PD with mucin pool |
| GCM06 | 29 | F | IIIB | Intestinal | PD |
| GCM07 | 43 | F | IIIB | Diffuse | Mucinous |
| GCM08 | 38 | F | IIIC | Diffuse | SRC |
| GCM09 | 47 | F | IIIC | Diffuse | SRC with mucin pool |
| GCM10 | 33 | M | IIIC | Diffuse | PD |
| GCM11 | 42 | M | IV | Intestinal | MD |
| GCM12 | 53 | F | IIIA | Diffuse | SRC |
| GCM13 | 38 | F | IV | Diffuse | PD with SRC |
| GCM14 | 56 | F | IIIB | Diffuse | SRC |
| GCM15 | 72 | M | IIIB | Intestinal | MD |

MD; moderate differentiated, PD; poorly differentiated, SRC; signet ring cell carcinoma

**Table S2. Tumor information for the patients enrolled in this study with metastatic gastric cance.**

| **PatientID** | **SampleID** | **Met Type** | **Met timing** | **Exposure to chemotherapy** | **Lauren classification** | **Purity*** | **Microsatellite instability status#** |
| --- | --- | --- | --- | --- | --- | --- | --- |
| GCM01 | GCM01_T | primary | synchronous | naïve | Intestinal | 0.5 | microsatellite stable |
| GCM01 | GCM01_liver | hematogenous | metachronous | naïve | Intestinal | 0.8 | microsatellite stable |
| GCM02 | GCM02_LN2 | LN | synchronous | naïve | Intestinal | 0.15 | microsatellite stable |
| GCM02 | GCM02_T1 | primary | synchronous | naïve | Intestinal | 0.25 | microsatellite stable |
| GCM02 | GCM02_T2 | primary | synchronous | naïve | Intestinal | 0.3 | microsatellite stable |
| GCM02 | GCM02_liver1 | hematogenous | metachronous | exposed | Intestinal | 0.7 | microsatellite stable |
| GCM02 | GCM02_liver2 | hematogenous | metachronous | exposed | Intestinal | 0.7 | microsatellite stable |
| GCM02 | GCM02_liver3 | hematogenous | metachronous | exposed | Intestinal | 0.6 | microsatellite stable |
| GCM03 | GCM03_LN4 | LN | synchronous | naïve | Intestinal | 0.5 | microsatellite stable |
| GCM03 | GCM03_LN6 | LN | synchronous | naïve | Intestinal | 0.6 | microsatellite stable |
| GCM03 | GCM03_T | primary | synchronous | naïve | Intestinal | 0.3 | microsatellite stable |
| GCM03 | GCM03_liver1 | hematogenous | metachronous | exposed | Intestinal | 0.7 | microsatellite stable |
| GCM03 | GCM03_liver2 | hematogenous | metachronous | exposed | Intestinal | 0.6 | microsatellite stable |
| GCM04 | GCM04_LN11 | LN | synchronous | naïve | Intestinal | 0.7 | microsatellite stable |
| GCM04 | GCM04_LND1 | LN | synchronous | naïve | Intestinal | 0.4 | microsatellite stable |
| GCM04 | GCM04_T1 | primary | synchronous | naïve | Intestinal | 0.45 | microsatellite stable |
| GCM04 | GCM04_T2 | primary | synchronous | naïve | Intestinal | 0.2 | microsatellite stable |
| GCM04 | GCM04_T3 | primary | synchronous | naïve | Intestinal | 0.3 | microsatellite stable |
| GCM04 | GCM04_lung | hematogenous | metachronous | exposed | Intestinal | 0.8 | microsatellite stable |
| GCM05 | GCM05_LND1 | LN | synchronous | naïve | Mixed | 0.1 | microsatellite stable |
| GCM05 | GCM05_T1 | primary | synchronous | naïve | Mixed | 0.15 | microsatellite stable |
| GCM05 | GCM05_T2 | primary | synchronous | naïve | Mixed | 0.1 | microsatellite stable |
| GCM05 | GCM05_T3 | primary | synchronous | naïve | Mixed | 0.1 | microsatellite stable |
| GCM05 | GCM05_perit1 | peritoneum | metachronous | exposed | Mixed | 0.05 | microsatellite stable |
| GCM05 | GCM05_perit2 | peritoneum | metachronous | exposed | Mixed | 0.05 | microsatellite stable |
| GCM06 | GCM06_LND1 | LN | synchronous | naïve | Intestinal | 0.1 | microsatellite stable |
| GCM06 | GCM06_LND2 | LN | synchronous | naïve | Intestinal | 0.05 | microsatellite stable |
| GCM06 | GCM06_T1a | primary | synchronous | naïve | Intestinal | 0.3 | microsatellite stable |
| GCM06 | GCM06_T1b | primary | synchronous | naïve | Intestinal | 0.1 | microsatellite stable |
| GCM06 | GCM06_T2 | primary | synchronous | naïve | Intestinal | 0.05 | microsatellite stable |
| GCM06 | GCM06_T3 | primary | synchronous | naïve | Intestinal | 0.05 | microsatellite stable |
| GCM06 | GCM06_ovary1 | ovary | metachronous | exposed | Intestinal | 0.15 | microsatellite stable |
| GCM06 | GCM06_ovary2 | ovary | metachronous | exposed | Intestinal | 0.3 | microsatellite stable |
| GCM07 | GCM07_LND4 | LN | metachronous | exposed | Diffuse | 0.4 | microsatellite stable |
| GCM07 | GCM07_LND1 | LN | synchronous | naïve | Diffuse | 0.4 | microsatellite stable |
| GCM07 | GCM07_T1a | primary | synchronous | naïve | Diffuse | 0.1 | microsatellite stable |
| GCM07 | GCM07_T1b | primary | synchronous | naïve | Diffuse | 0.6 | microsatellite stable |
| GCM07 | GCM07_T2 | primary | synchronous | naïve | Diffuse | 0.6 | microsatellite stable |
| GCM07 | GCM07_T3 | primary | synchronous | naïve | Diffuse | 0.2 | microsatellite stable |
| GCM07 | GCM07_ovary | ovary | metachronous | exposed | Diffuse | 0.6 | microsatellite stable |
| GCM08 | GCM08_LND1 | LN | synchronous | naïve | Diffuse | 0.4 | microsatellite stable |
| GCM08 | GCM08_T1 | primary | synchronous | naïve | Diffuse | 0.9 | microsatellite stable |
| GCM08 | GCM08_T2 | primary | synchronous | naïve | Diffuse | 0.5 | microsatellite stable |
| GCM08 | GCM08_T3 | primary | synchronous | naïve | Diffuse | 0.3 | microsatellite stable |
| GCM08 | GCM08_ovary1 | ovary | metachronous | exposed | Diffuse | 0.15 | microsatellite stable |
| GCM08 | GCM08_ovary2 | ovary | metachronous | exposed | Diffuse | 0.15 | microsatellite stable |
| GCM09 | GCM09_LN10 | LN | synchronous | naïve | Diffuse | 0.4 | microsatellite stable |
| GCM09 | GCM09_LN3 | LN | synchronous | naïve | Diffuse | 0.35 | microsatellite stable |
| GCM09 | GCM09_T1 | primary | synchronous | naïve | Diffuse | 0.8 | microsatellite stable |
| GCM09 | GCM09_T2 | primary | synchronous | naïve | Diffuse | 0.5 | microsatellite stable |
| GCM09 | GCM09_T3 | primary | synchronous | naïve | Diffuse | 0.4 | microsatellite stable |
| GCM09 | GCM09_perit | peritoneum | metachronous | exposed | Diffuse | 0.05 | microsatellite stable |
| GCM09 | GCM09_ovary1 | ovary | metachronous | exposed | Diffuse | 0.4 | microsatellite stable |
| GCM09 | GCM09_ovary2 | ovary | metachronous | exposed | Diffuse | 0.3 | microsatellite stable |
| GCM10 | GCM10_LN16 | LN | synchronous | exposed | Diffuse | 0.2 | microsatellite stable |
| GCM10 | GCM10_LN1 | LN | synchronous | exposed | Diffuse | 0.4 | microsatellite stable |
| GCM10 | GCM10_LN3 | LN | synchronous | exposed | Diffuse | 0.2 | microsatellite stable |
| GCM10 | GCM10_LN4 | LN | synchronous | exposed | Diffuse | 0.4 | microsatellite stable |
| GCM10 | GCM10_LN8 | LN | synchronous | exposed | Diffuse | 0.3 | microsatellite stable |
| GCM10 | GCM10_LN9 | LN | synchronous | exposed | Diffuse | 0.3 | microsatellite stable |
| GCM10 | GCM10_T1a | primary | synchronous | exposed | Diffuse | 0.3 | microsatellite stable |
| GCM10 | GCM10_T1b | primary | synchronous | exposed | Diffuse | 0.05 | microsatellite stable |
| GCM10 | GCM10_T2 | primary | synchronous | exposed | Diffuse | 0.01 | microsatellite stable |
| GCM10 | GCM10_T3 | primary | synchronous | exposed | Diffuse | 0.05 | microsatellite stable |
| GCM10 | GCM10_liver | hematogenous | synchronous | exposed | Diffuse | 0.4 | microsatellite stable |
| GCM11 | GCM11_LN14v | LN | synchronous | naïve | Intestinal | 0.3 | microsatellite stable |
| GCM11 | GCM11_LN16 | LN | synchronous | naïve | Intestinal | 0.3 | microsatellite stable |
| GCM11 | GCM11_LN1 | LN | synchronous | naïve | Intestinal | 0.3 | microsatellite stable |
| GCM11 | GCM11_LN4 | LN | synchronous | naïve | Intestinal | 0.7 | microsatellite stable |
| GCM11 | GCM11_LN7 | LN | synchronous | naïve | Intestinal | 0.4 | microsatellite stable |
| GCM11 | GCM11_LN8 | LN | synchronous | naïve | Intestinal | 0.3 | microsatellite stable |
| GCM11 | GCM11_LN9 | LN | synchronous | naïve | Intestinal | 0.25 | microsatellite stable |
| GCM11 | GCM11_T1a | primary | synchronous | naïve | Intestinal | 0.4 | microsatellite stable |
| GCM11 | GCM11_T1b | primary | synchronous | naïve | Intestinal | 0.5 | microsatellite stable |
| GCM11 | GCM11_T2 | primary | synchronous | naïve | Intestinal | 0.4 | microsatellite stable |
| GCM11 | GCM11_T3 | primary | synchronous | naïve | Intestinal | 0.4 | microsatellite stable |
| GCM12 | GCM12_LN | LN | synchronous | naïve | Diffuse | 0.05 | microsatellite stable |
| GCM12 | GCM12_T1a | primary | synchronous | naïve | Diffuse | 0.1 | microsatellite stable |
| GCM12 | GCM12_T1b | primary | synchronous | naïve | Diffuse | 0.1 | microsatellite stable |
| GCM12 | GCM12_T2 | primary | synchronous | naïve | Diffuse | 0.05 | microsatellite stable |
| GCM12 | GCM12_perit | peritoneum | synchronous | naïve | Diffuse | 0.02 | microsatellite stable |
| GCM12 | GCM12_ovary1 | ovary | synchronous | naïve | Diffuse | 0.3 | microsatellite stable |
| GCM12 | GCM12_ovary2 | ovary | synchronous | naïve | Diffuse | 0.2 | microsatellite stable |
| GCM13 | GCM13_LND1 | LN | synchronous | naïve | Diffuse | 0.1 | microsatellite stable |
| GCM13 | GCM13_LND2 | LN | synchronous | naïve | Diffuse | 0.22 | microsatellite stable |
| GCM13 | GCM13_T1 | primary | synchronous | naïve | Diffuse | 0.4 | microsatellite stable |
| GCM13 | GCM13_T2 | primary | synchronous | naïve | Diffuse | 0.2 | microsatellite stable |
| GCM13 | GCM13_perit | peritoneum | synchronous | naïve | Diffuse | 0.3 | microsatellite stable |
| GCM13 | GCM13_ovary1 | ovary | synchronous | naïve | Diffuse | 0.05 | microsatellite stable |
| GCM13 | GCM13_ovary2 | ovary | synchronous | naïve | Diffuse | 0.3 | microsatellite stable |
| GCM14 | GCM14_T1 | primary | synchronous | naïve | Diffuse | 0.27 | microsatellite stable |
| GCM14 | GCM14_T2 | primary | synchronous | naïve | Diffuse | 0.02 | microsatellite stable |
| GCM14 | GCM14_perit | peritoneum | metachronous | exposed | Diffuse | 0.9 | microsatellite stable |
| GCM14 | GCM14_ovary1 | ovary | metachronous | exposed | Diffuse | 0.15 | microsatellite stable |
| GCM14 | GCM14_ovary2 | ovary | metachronous | exposed | Diffuse | 0.2 | microsatellite stable |
| GCM15 | GCM15_T1 | primary | synchronous | naïve | Intestinal | 0.32 | microsatellite stable |
| GCM15 | GCM15_T2 | primary | synchronous | naïve | Intestinal | 0.25 | microsatellite stable |
| GCM15 | GCM15_T3 | primary | synchronous | naïve | Intestinal | 0.1 | microsatellite stable |
| GCM15 | GCM15_liver | hematogenous | metachronous | exposed | Intestinal | 0.3 | microsatellite stable |

* estimated by ASCAT

# estimated by MSI Sensor

**Table S3. Germline DNA whole exome sequencing statistics.**

| **Sample ID** | **N reads** | **Percentage of reads** | | **Percentage coverage of target regions (more than 20X)** | **Average coverage on target** | **Mean insert size (bp)** | **Percentage of properly paired reads** | **Number of variants** | | | **Ts/Tv** | **% GC** |
| --- | --- | --- | --- | --- | --- | --- | --- | --- | --- | --- | --- | --- |
|  |  | **Mapped** | **Duplicated** |  |  |  |  | **Total** | **SNV** | **Indel** |  |  |
| **GCM01_normal** | 180.6 M | 99.80% | 22.70% | 80.00% | 100 | 123 | 99.20% | 54636 | 50896 | 3740 | 2.52 | 53% |
| **GCM02_normal** | 199.2 M | 99.80% | 16.40% | 77.20% | 135 | 151 | 99.00% | 56651 | 51780 | 4871 | 2.45 | 52% |
| **GCM03_normal** | 238.0 M | 99.90% | 51.40% | 81.50% | 75 | 104 | 99.20% | 55051 | 51192 | 3859 | 2.51 | 54% |
| **GCM04_normal** | 219.1 M | 99.80% | 15.30% | 76.10% | 152 | 158 | 98.90% | 56522 | 51457 | 5065 | 2.46 | 52% |
| **GCM05_normal** | 193.8 M | 99.80% | 15.30% | 79.30% | 136 | 150 | 98.90% | 55919 | 51265 | 4654 | 2.47 | 53% |
| **GCM06_normal** | 245.6 M | 99.80% | 30.40% | 82.70% | 124 | 120 | 99.20% | 54640 | 50770 | 3870 | 2.5 | 53% |
| **GCM07_normal** | 224.7 M | 99.70% | 17.80% | 78.60% | 153 | 153 | 98.90% | 56261 | 51256 | 5005 | 2.46 | 52% |
| **GCM08_normal** | 219.1 M | 99.90% | 16.10% | 80.50% | 153 | 148 | 99.10% | 56220 | 51729 | 4491 | 2.47 | 52% |
| **GCM09_normal** | 205.2 M | 99.70% | 14.10% | 77.00% | 147 | 158 | 98.60% | 56207 | 51299 | 4908 | 2.45 | 53% |
| **GCM10_normal** | 190.0 M | 99.80% | 47.80% | 78.80% | 62 | 104 | 98.80% | 55431 | 51744 | 3687 | 2.56 | 55% |
| **GCM11_normal** | 212.2 M | 99.80% | 13.60% | 74.80% | 158 | 181 | 98.80% | 56910 | 51823 | 5087 | 2.47 | 52% |
| **GCM12_normal** | 224.7 M | 99.70% | 15.80% | 80.10% | 161 | 154 | 98.80% | 56234 | 50991 | 5259 | 2.46 | 52% |
| **GCM13_normal** | 210.3 M | 99.80% | 14.90% | 77.00% | 158 | 179 | 98.80% | 56464 | 50990 | 5491 | 2.44 | 52% |
| **GCM14_normal** | 195.4 M | 99.90% | 35.50% | 81.10% | 83 | 108 | 99.00% | 54306 | 49686 | 4630 | 2.47 | 55% |
| **GCM15_normal** | 230.8 M | 99.90% | 19.60% | 78.90% | 148 | 143 | 99.10% | 55450 | 50573 | 4889 | 2.46 | 53% |

**Table S4. Somatic DNA whole exome sequencing statistics.**

| **Sample ID** | **N reads** | **Percentage of reads** | | **Percentage coverage of target regions (more than 20X)** | **Average coverage on target** | **Mean insert size (bp)** | **Percentage of properly paired reads** | **Number of variants** | | | **% GC** |
| --- | --- | --- | --- | --- | --- | --- | --- | --- | --- | --- | --- |
|  |  | **Mapped** | **Duplicated** |  |  |  |  | **Total** | **SNV** | **Indel** |  |
| **GCM01_T** | 371.4 M | 99.50% | 25.90% | 72.60% | 193 | 137 | 98.40% | 72999 | 73001 | 1204 | 54% |
| **GCM01_liver** | 211.8 M | 99.90% | 22.80% | 80.60% | 120 | 125 | 99.30% | 18545 | 18548 | 109 | 54% |
| **GCM02_LND1** | 198.2 M | 99.90% | 15.40% | 75.10% | 143 | 175 | 98.90% | 7903 | 7904 | 34 | 52% |
| **GCM02_T1** | 215.0 M | 99.80% | 17.70% | 79.70% | 147 | 149 | 99.10% | 17890 | 17892 | 98 | 53% |
| **GCM02_T2** | 243.7 M | 99.80% | 16.70% | 78.30% | 169 | 154 | 98.90% | 12956 | 12957 | 102 | 53% |
| **GCM02_liver1** | 194.7 M | 99.90% | 44.20% | 81.80% | 71 | 106 | 99.30% | 18207 | 18217 | 139 | 56% |
| **GCM02_liver2** | 255.3 M | 99.80% | 30.20% | 79.40% | 130 | 128 | 99.20% | 20401 | 20407 | 156 | 55% |
| **GCM02_liver3** | 199.9 M | 99.80% | 53.80% | 83.10% | 60 | 102 | 99.20% | 17615 | 17632 | 123 | 56% |
| **GCM03_LN4** | 197.1 M | 99.70% | 60.00% | 79.20% | 49 | 102 | 99.00% | 21240 | 21246 | 98 | 56% |
| **GCM03_LN6** | 197.8 M | 99.90% | 46.60% | 83.50% | 71 | 107 | 99.10% | 22361 | 22370 | 90 | 55% |
| **GCM03_T** | 194.7 M | 99.60% | 37.50% | 77.70% | 78 | 111 | 99.00% | 22265 | 22272 | 176 | 55% |
| **GCM03_liver1** | 274.7 M | 98.70% | 23.60% | 78.20% | 171 | 153 | 97.70% | 40908 | 40939 | 137 | 54% |
| **GCM03_liver2** | 279.9 M | 99.70% | 22.70% | 76.90% | 178 | 156 | 98.70% | 10969 | 10991 | 118 | 54% |
| **GCM04_LND1** | 250.5 M | 99.80% | 21.60% | 77.90% | 161 | 152 | 98.90% | 18819 | 18820 | 157 | 54% |
| **GCM04_LND2** | 218.4 M | 99.90% | 20.30% | 78.20% | 146 | 156 | 99.00% | 12275 | 12275 | 116 | 53% |
| **GCM04_T1** | 215.0 M | 99.80% | 19.30% | 77.40% | 146 | 160 | 98.80% | 11841 | 11841 | 68 | 54% |
| **GCM04_T2** | 251.3 M | 99.80% | 21.20% | 76.00% | 168 | 169 | 98.70% | 11831 | 11832 | 50 | 53% |
| **GCM04_T3** | 240.7 M | 99.90% | 19.70% | 78.60% | 162 | 154 | 98.70% | 13287 | 13291 | 179 | 55% |
| **GCM04_lung** | 234.9 M | 99.90% | 35.20% | 82.30% | 106 | 114 | 99.20% | 26225 | 26230 | 133 | 54% |
| **GCM05_LND1** | 210.6 M | 99.70% | 20.80% | 76.90% | 144 | 171 | 98.60% | 15942 | 15942 | 30 | 52% |
| **GCM05_T1** | 265.0 M | 99.80% | 22.00% | 75.10% | 163 | 151 | 98.90% | 9778 | 9778 | 135 | 53% |
| **GCM05_T2** | 250.9 M | 99.90% | 22.40% | 74.80% | 157 | 157 | 98.90% | 6713 | 6713 | 62 | 53% |
| **GCM05_T3** | 261.9 M | 99.90% | 22.00% | 77.60% | 172 | 159 | 98.90% | 11529 | 11530 | 69 | 53% |
| **GCM05_perit1** | 223.8 M | 99.20% | 19.80% | 78.50% | 148 | 153 | 98.20% | 13195 | 13195 | 61 | 54% |
| **GCM05_perit2** | 278.8 M | 99.20% | 22.10% | 75.50% | 181 | 167 | 98.20% | 11606 | 11606 | 58 | 53% |
| **GCM06_LND1** | 263.8 M | 99.90% | 22.30% | 77.10% | 172 | 160 | 98.80% | 12293 | 12294 | 48 | 53% |
| **GCM06_LND2** | 186.8 M | 99.90% | 16.70% | 78.30% | 131 | 155 | 98.90% | 15100 | 15101 | 55 | 53% |
| **GCM06_T1a** | 283.2 M | 99.90% | 38.70% | 79.60% | 119 | 117 | 99.30% | 22758 | 22758 | 160 | 55% |
| **GCM06_T1b** | 181.0 M | 99.80% | 52.50% | 79.00% | 53 | 101 | 98.80% | 15734 | 15739 | 55 | 56% |
| **GCM06_T2** | 274.9 M | 99.90% | 53.00% | 79.40% | 84 | 110 | 99.10% | 19753 | 19757 | 122 | 56% |
| **GCM06_T3** | 214.7 M | 99.90% | 42.60% | 80.60% | 81 | 109 | 99.00% | 19797 | 19801 | 106 | 55% |
| **GCM06_ovary1** | 194.0 M | 99.30% | 15.70% | 78.40% | 138 | 160 | 98.30% | 16751 | 16751 | 72 | 53% |
| **GCM06_ovary2** | 181.4 M | 99.90% | 14.80% | 73.70% | 124 | 161 | 98.90% | 11515 | 11519 | 37 | 52% |
| **GCM07_LND1** | 210.7 M | 99.80% | 17.10% | 75.80% | 143 | 160 | 99.00% | 16927 | 16927 | 65 | 52% |
| **GCM07_T1a** | 193.4 M | 99.90% | 21.40% | 81.50% | 115 | 128 | 99.30% | 23253 | 23253 | 78 | 54% |
| **GCM07_T1b** | 194.6 M | 99.80% | 16.40% | 79.30% | 131 | 144 | 99.10% | 15062 | 15062 | 79 | 53% |
| **GCM07_T2** | 197.1 M | 99.90% | 15.00% | 78.40% | 135 | 147 | 99.10% | 14350 | 14350 | 82 | 53% |
| **GCM07_T3** | 196.1 M | 99.90% | 17.90% | 77.80% | 125 | 140 | 99.20% | 25331 | 25331 | 95 | 54% |
| **GCM07_LND4** | 213.5 M | 99.70% | 16.10% | 75.20% | 147 | 161 | 98.80% | 10257 | 10257 | 40 | 52% |
| **GCM07_ovary** | 182.5 M | 99.80% | 13.70% | 76.20% | 133 | 166 | 98.90% | 8924 | 8924 | 56 | 52% |
| **GCM08_LND1** | 183.6 M | 99.90% | 14.10% | 77.20% | 128 | 151 | 99.00% | 24153 | 24153 | 66 | 53% |
| **GCM08_T1** | 184.5 M | 99.90% | 14.10% | 77.90% | 125 | 144 | 99.10% | 18620 | 18620 | 104 | 53% |
| **GCM08_T2** | 188.2 M | 99.80% | 14.80% | 77.40% | 131 | 152 | 99.00% | 16756 | 16756 | 80 | 53% |
| **GCM08_T3** | 191.0 M | 99.90% | 14.60% | 78.60% | 133 | 148 | 99.10% | 17495 | 17496 | 84 | 53% |
| **GCM08_ovary1** | 198.9 M | 99.80% | 13.10% | 74.30% | 145 | 172 | 98.50% | 8622 | 8622 | 40 | 53% |
| **GCM08_ovary2** | 221.3 M | 99.80% | 13.20% | 72.90% | 160 | 178 | 98.50% | 9510 | 9510 | 41 | 52% |
| **GCM09_LND1** | 197.5 M | 99.80% | 19.30% | 71.90% | 131 | 181 | 98.50% | 22316 | 22316 | 26 | 53% |
| **GCM09_LND2** | 216.3 M | 99.80% | 16.40% | 73.40% | 154 | 185 | 98.60% | 31619 | 31619 | 25 | 52% |
| **GCM09_T1** | 215.9 M | 99.90% | 14.10% | 77.00% | 153 | 157 | 98.90% | 14068 | 14069 | 53 | 53% |
| **GCM09_T2** | 208.5 M | 99.80% | 14.10% | 75.30% | 144 | 155 | 98.90% | 21744 | 21747 | 71 | 53% |
| **GCM09_T3** | 198.0 M | 99.90% | 12.90% | 73.30% | 139 | 163 | 98.90% | 8273 | 8274 | 28 | 52% |
| **GCM09_ovary1** | 206.6 M | 99.80% | 16.30% | 72.30% | 143 | 179 | 98.60% | 11712 | 11713 | 23 | 52% |
| **GCM09_ovary2** | 230.4 M | 99.60% | 17.80% | 72.40% | 158 | 184 | 98.50% | 6214 | 6214 | 24 | 51% |
| **GCM09_perit** | 285.4 M | 99.80% | 17.40% | 71.80% | 200 | 192 | 98.60% | 2889 | 2890 | 21 | 52% |
| **GCM10_LN12** | 218.8 M | 99.80% | 21.50% | 78.10% | 129 | 134 | 99.10% | 22200 | 22205 | 163 | 54% |
| **GCM10_LN1** | 383.3 M | 99.80% | 26.90% | 73.00% | 199 | 137 | 98.90% | 69977 | 69977 | 1068 | 55% |
| **GCM10_LN3** | 231.6 M | 99.80% | 26.40% | 79.30% | 125 | 128 | 99.20% | 24969 | 24971 | 181 | 55% |
| **GCM10_LN4** | 215.5 M | 99.90% | 34.00% | 80.70% | 97 | 114 | 99.20% | 26426 | 26431 | 192 | 55% |
| **GCM10_LN8** | 234.5 M | 99.80% | 22.60% | 78.60% | 134 | 131 | 99.10% | 23793 | 23798 | 152 | 55% |
| **GCM10_LN9** | 274.9 M | 99.80% | 23.50% | 76.10% | 155 | 135 | 99.00% | 25428 | 25433 | 162 | 55% |
| **GCM10_T1a** | 209.7 M | 99.70% | 28.70% | 81.20% | 106 | 120 | 99.00% | 27769 | 27776 | 162 | 56% |
| **GCM10_T1b** | 223.3 M | 99.50% | 23.00% | 76.10% | 121 | 128 | 98.70% | 27085 | 27090 | 199 | 55% |
| **GCM10_T2** | 195.3 M | 99.90% | 24.00% | 77.70% | 104 | 123 | 99.10% | 23629 | 23632 | 134 | 55% |
| **GCM10_T3** | 267.1 M | 99.90% | 24.30% | 78.00% | 156 | 139 | 99.20% | 24778 | 24782 | 163 | 56% |
| **GCM10_liver** | 199.3 M | 99.80% | 24.30% | 80.10% | 106 | 120 | 99.30% | 37034 | 37039 | 167 | 55% |
| **GCM11_LN10** | 282.2 M | 99.80% | 20.60% | 76.00% | 187 | 165 | 98.90% | 12373 | 12373 | 48 | 52% |
| **GCM11_LN11** | 182.2 M | 99.00% | 15.90% | 74.80% | 124 | 161 | 98.00% | 16980 | 16980 | 74 | 53% |
| **GCM11_LN14v** | 221.2 M | 99.80% | 20.20% | 76.10% | 143 | 156 | 98.90% | 21130 | 21130 | 153 | 53% |
| **GCM11_LN16** | 191.3 M | 99.80% | 18.20% | 76.20% | 128 | 158 | 98.80% | 24518 | 24519 | 100 | 53% |
| **GCM11_LN1** | 257.5 M | 99.30% | 17.60% | 77.50% | 172 | 152 | 98.40% | 16500 | 16500 | 103 | 52% |
| **GCM11_LN4** | 206.2 M | 99.90% | 17.60% | 76.70% | 138 | 154 | 99.00% | 25737 | 25737 | 84 | 52% |
| **GCM11_LN9** | 270.9 M | 99.80% | 18.30% | 77.40% | 184 | 158 | 98.90% | 19697 | 19698 | 66 | 52% |
| **GCM11_T1a** | 213.4 M | 99.30% | 15.20% | 76.20% | 151 | 164 | 98.20% | 9824 | 9825 | 41 | 53% |
| **GCM11_T1b** | 222.1 M | 99.30% | 15.60% | 76.40% | 160 | 170 | 98.20% | 10081 | 10081 | 36 | 52% |
| **GCM11_T2** | 252.5 M | 99.40% | 20.40% | 72.90% | 168 | 185 | 98.10% | 20116 | 20116 | 18 | 51% |
| **GCM11_T3** | 229.5 M | 98.80% | 17.20% | 73.70% | 160 | 184 | 97.70% | 9284 | 9284 | 17 | 51% |
| **GCM012_LN** | 207.5 M | 99.80% | 17.80% | 79.70% | 149 | 162 | 98.50% | 2647 | 2647 | 38 | 55% |
| **GCM012_T1a** | 218.7 M | 99.10% | 15.60% | 78.30% | 153 | 154 | 98.30% | 7704 | 7704 | 37 | 54% |
| **GCM012_T1b** | 262.1 M | 98.70% | 17.20% | 77.50% | 187 | 171 | 97.60% | 2728 | 2728 | 52 | 53% |
| **GCM012_T2** | 247.1 M | 98.70% | 15.50% | 77.80% | 182 | 174 | 97.60% | 3387 | 3387 | 56 | 53% |
| **GCM012_ovary1** | 242.2 M | 99.80% | 15.00% | 80.10% | 173 | 150 | 97.60% | 3135 | 3135 | 61 | 56% |
| **GCM012_ovary2** | 245.9 M | 99.90% | 13.60% | 79.30% | 181 | 156 | 98.60% | 6188 | 6190 | 82 | 55% |
| **GCM012_perit** | 257.6 M | 99.20% | 15.20% | 76.60% | 189 | 176 | 98.10% | 2105 | 2105 | 29 | 53% |
| **GCM013_LND1** | 277.5 M | 99.80% | 17.30% | 75.10% | 201 | 189 | 98.70% | 6515 | 6515 | 13 | 51% |
| **GCM013_LND2** | 218.7 M | 99.90% | 14.90% | 75.90% | 167 | 194 | 98.70% | 1728 | 1728 | 5 | 52% |
| **GCM013_T1** | 286.1 M | 99.80% | 18.30% | 76.30% | 205 | 178 | 98.70% | 1295 | 1295 | 18 | 52% |
| **GCM013_T2** | 209.3 M | 99.80% | 15.40% | 77.30% | 157 | 178 | 98.80% | 1236 | 1236 | 10 | 51% |
| **GCM013_ovary1** | 206.8 M | 99.90% | 13.40% | 77.30% | 156 | 172 | 98.70% | 6415 | 6415 | 28 | 52% |
| **GCM013_ovary2** | 203.0 M | 99.80% | 14.10% | 76.30% | 153 | 181 | 98.70% | 3249 | 3249 | 18 | 52% |
| **GCM013_perit** | 221.7 M | 99.50% | 13.80% | 75.70% | 171 | 198 | 98.40% | 1724 | 1724 | 11 | 51% |
| **GCM014_T1** | 199.3 M | 99.90% | 34.00% | 76.60% | 81 | 108 | 99.20% | 32493 | 32499 | 250 | 55% |
| **GCM014_T2** | 197.9 M | 99.90% | 29.80% | 74.40% | 86 | 111 | 99.20% | 42823 | 42824 | 256 | 54% |
| **GCM014_ovary1** | 228.0 M | 99.80% | 21.60% | 74.80% | 148 | 167 | 97.30% | 7552 | 7553 | 68 | 52% |
| **GCM014_ovary2** | 196.1 M | 99.90% | 14.90% | 79.40% | 142 | 156 | 98.90% | 10304 | 10304 | 164 | 53% |
| **GCM14_perit** | 242.5 M | 99.90% | 16.80% | 76.40% | 174 | 174 | 98.90% | 4968 | 4970 | 48 | 52% |
| **GCM015_T1** | 246.6 M | 99.80% | 20.30% | 78.30% | 152 | 139 | 99.10% | 22274 | 22274 | 408 | 54% |
| **GCM015_T2** | 212.0 M | 99.80% | 16.30% | 80.50% | 149 | 150 | 99.00% | 16795 | 16795 | 320 | 52% |
| **GCM015_T3** | 215.4 M | 99.80% | 17.80% | 79.60% | 145 | 147 | 98.90% | 14591 | 14592 | 274 | 52% |
| **GCM015_liver** | 259.5 M | 99.90% | 23.80% | 74.80% | 136 | 126 | 99.30% | 40298 | 40302 | 422 | 54% |

**Table S5. Inferred phylogeny and migration history determined using Treeomics, MEDDIC2, consensus, and MACHINA.**

| **PatientID** | **Treeomics** | **MEDICC2** | **Consensus** | **MACHINA** |
| --- | --- | --- | --- | --- |
| GCM01 | Branched | Branched | Branched | mPS |
| GCM02 | Branched | Branched | Branched | pPS |
| GCM03 | Branched | Branched | Branched | pPS |
| GCM04 | Branched | Branched | Branched | pS |
| GCM05 | Branched | Branched | Branched | pS |
| GCM06 | Branched | Branched | Branched | pPS |
| GCM07 | Branched | Branched | Branched | pS |
| GCM08 | Branched | Diaspora | Diaspora | pPS |
| GCM09 | Branched | Branched | Branched | pPS |
| GCM10 | Diaspora | Diaspora | Diaspora | pPS |
| GCM11 | Branched | Branched | Branched | pPS |
| GCM12 | Branched | Branched | Branched | pPS |
| GCM13 | Branched | Branched | Branched | pPS |
| GCM14 | Branched | Branched | Branched | pS |
| GCM15 | Branched | Branched | Branched | pPS |

m; monoclonal, p; polyclonal; P; pararrel, S; single source seeding
